# Bacteriophages Synergize with the Gut Microbial Community To Combat *Salmonella*

**DOI:** 10.1128/mSystems.00119-18

**Published:** 2018-10-02

**Authors:** Yue O. O. Hu, Luisa W. Hugerth, Carina Bengtsson, Arlisa Alisjahbana, Maike Seifert, Anaga Kamal, Åsa Sjöling, Tore Midtvedt, Elisabeth Norin, Juan Du, Lars Engstrand

**Affiliations:** a Department of Microbiology, Tumor and Cell Biology, Centre for Translational Microbiome Research (CTMR), Karolinska Institutet, Stockholm, Sweden; b Science for Life Laboratory, Stockholm, Sweden; c Department of Microbiology, Tumor and Cell Biology, Karolinska Institutet, Stockholm, Sweden; University of California, Irvine

**Keywords:** anaerobically cultivated human intestinal microflora (ACHIM), microbiota, *Salmonella*, antibiotic resistance, bacteriophages

## Abstract

*Salmonella* infection is one of the main causes of food-borne diarrheal diseases worldwide. Although most *Salmonella* infections can be cleared without treatment, some cause serious illnesses that require antibiotic treatment. In view of the growing emergence of antibiotic-resistant *Salmonella* strains, novel treatments are increasingly required. Furthermore, there is a striking paucity of data on how a balanced human gut microbiota responds to *Salmonella* infection. This study aimed to evaluate whether a balanced gut microbiota protects against *Salmonella* growth and to compare two antimicrobial approaches for managing *Salmonella* infection: bacteriophage (phage) treatment and antibiotic treatment. Anaerobically cultivated human intestinal microflora (ACHIM) is a feasible model for the human gut microbiota and naturally inhibits *Salmonella* infection. By mimicking *Salmonella* infection *in vitro* using ACHIM, we observed a large reduction of *Salmonella* growth by the ACHIM itself. Treatments with phage and antibiotic further inhibited *Salmonella* growth. However, phage treatment had less impact on the nontargeted bacteria in ACHIM than the antibiotic treatment did. Phage treatment has high specificity when combating *Salmonella* infection and offers a noninvasive alternative to antibiotic treatment.

**IMPORTANCE** Antibiotic-resistant bacteria are a global threat. Therefore, alternative approaches for combatting bacteria, especially antibiotic-resistant bacteria, are urgently needed. Using a human gut microbiota model, we demonstrate that bacteriophages (phages) are able to substantially decrease pathogenic *Salmonella* without perturbing the microbiota. Conversely, antibiotic treatment leads to the eradication of close to all commensal bacteria, leaving only antibiotic-resistant bacteria. An unbalanced microbiota has been linked to many diseases both in the gastrointestinal tract or “nonintestinal” diseases. In our study, we show that the microbiota provides a protective effect against *Salmonella*. Since phage treatment preserves the healthy gut microbiota, it is a feasible superior alternative to antibiotic treatment. Furthermore, when combating infections caused by pathogenic bacteria, gut microbiota should be considered.

**Author Video**: An author video summary of this article is available.

## Abstract (precis)

Antibiotic-resistant bacteria are a global threat. Therefore, alternative approaches for combatting bacteria, especially antibiotic-resistant bacteria, are urgently needed. Using a human gut microbiota model, we demonstrate that bacteriophages (phages) are able to substantially decrease pathogenic *Salmonella* without perturbing the microbiota. Conversely, antibiotic treatment leads to the eradication of close to all commensal bacteria, leaving only antibiotic-resistant bacteria. An unbalanced microbiota has been linked to many diseases both in the gastrointestinal tract or “nonintestinal” diseases. In our study, we show that the microbiota provides a protective effect against *Salmonella*. Since phage treatment preserves the healthy gut microbiota, it is a feasible superior alternative to antibiotic treatment. Furthermore, when combating infections caused by pathogenic bacteria, gut microbiota should be considered.

## INTRODUCTION

The human gastrointestinal (GI) tract is colonized by an abundant and diverse microbiota. Alterations of the microbiota, such as through antibiotic treatment, may contribute to many chronic and degenerative diseases, including Crohn’s disease, ulcerative colitis, rheumatoid arthritis, irritable bowel syndrome, inflammatory bowel disease, and other “intestinal dysbioses” (1–4). Additionally, the human microbiota has increasingly been associated with “nonintestinal” diseases such as certain forms of cancer, aging, obesity, diabetes, and various neurological disorders (5, 6). A balanced gut microbiota not only provides a functional metabolic cycle but also trains the immune system in detecting pathogens and combats abnormal conditions such as dysbiosis (7–13). In addition, the gut microbiota is also able to influence immune therapy responses and therapeutic efficacy against tumors (14, 15). Thus, it is critical to have a thorough understanding of the gut microbiota and how various factors influence it, especially when developing science-based approaches for various health benefits.

Reaching that goal, however, is complicated by the lack of good culture models and tools that are required for the comprehensive study of the gut microbiota. We previously established an anaerobic *in vitro* gut microbiota culture named anaerobically cultivated human intestinal microflora (ACHIM) (16, 17). ACHIM contains most of the gut microbiota community from a healthy adult donor. This culture is maintained by weekly passages, is stable at the species level, and is free of pathogens (16, 17) (patent application WO 2013/053836A). In this study, we used ACHIM to advance our understanding of how the gut microbiota reacts or responds to pathogens.

*Salmonella* infection is one of the main global causes of diarrheal diseases and the third most common cause of diarrheal mortality (18). Typically, *Salmonella* infection in immunocompetent adults causes a self-limiting diarrheal disease, which resolves within 5 to 7 days. Antibiotics are generally not prescribed for treating salmonellosis, unless it becomes critical for patient survival or recovery. Hence, we hypothesize that in addition to a competent human immune system, a balanced gut microbiota might be essential for pathogen clearance.

The increasing emergence of *Salmonella* strains resistant to many currently available antibiotics highlights the need to identify novel alternatives and complements to antibiotic treatment for *Salmonella* infections (19–22). Lytic bacteriophages (phages) may provide one such approach. Phage treatment originated with the discovery of lytic phages, i.e., viruses that selectively infect and kill bacteria. It has been used for detecting different bacteria in infections and is the standard treatment for bacterial infections in several Eastern European countries (23–25). Due to the spread of multidrug-resistant (MDR) bacteria, interest in phage treatment is increasing, and phage treatment has been tested in clinical trials against many infections, such as *Pseudomonas*, *Escherichia coli, Shigella*, and *Staphylococcus* (26–30). Phage treatment has multiple advantages compared to antibiotics. These advantages include high specificity, low inherent toxicity, minimal disruption of surrounding tissues/normal flora, and low risk for resistance induction. Further, phage treatment allows for automated dosing, possesses the ability to clear biofilm, and can multiply at the infection site (25, 31, 32). The efficacy of phage therapy is likely to be dictated by the life cycle of phages and by the targeted bacterial species, so a phage cocktail may be required for maximized outcomes (23, 29, 31). In addition, phages could be subject to immune system detection with subsequent clearance. However, phage treatment has displayed minimal adverse effects both preclinically and clinically (27, 30, 33). Therefore, phage treatment exhibits significant potential as an alternative or replacement treatment for bacterial infections, especially when overcoming antibiotic-resistant bacteria (23, 34).

Phages have been utilized to treat *Salmonella* in the food industry and to control *Salmonella* in human and pet foods as well as on various surfaces (35–42). However, much less is known regarding how phage treatment affects the human microbiota following *Salmonella* infection (35–42). In this study, by using Salmonella serovar Enteriditis as a target pathogenic organism model and *Salmonella*-specific phages as treatment, we evaluated whether phages can be used as a replacement for antibiotic treatment. Further, we investigated how the gut microbiota changes in the presence or absence of antibiotic and lytic phages.

## RESULTS

### ACHIM reduces *Salmonella* growth.

**FIG 1:**
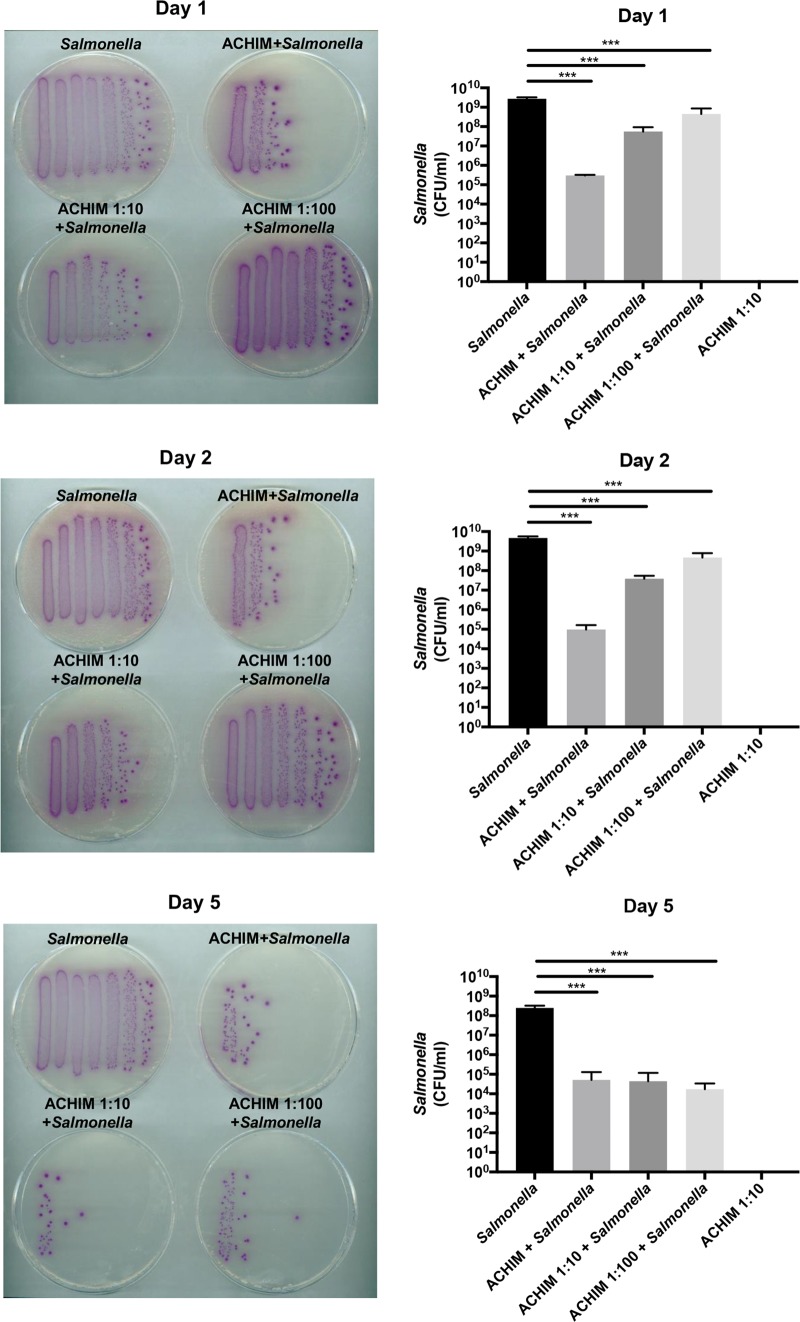
ACHIM decreases *Salmonella* growth. (Left) *Salmonella* growth on *Salmonella*-selective agar plates imaged on days 1, 2, and 5. The treatment conditions are indicated above the plates, and each bacterial streak/column reflects one concentration of a serially diluted culture. (Right) *Salmonella* quantification (CFU per milliliter) for control and treatment conditions in a log scale from each experimental day. Values that are statistically significantly different (*P* < 0.001) for each ACHIM concentration group and ACHIM-plus-*Salmonella* group by one-tailed *t* test analysis are indicated by bars and three asterisks. Values are means plus standard deviations (SD) (error bars) from three experiments.

Since ACHIM provides an ideal test platform and a proxy for a balanced human gut microbiota, we mimicked infection by adding Salmonella serovar Enteriditis (Salmonella) to ACHIM and performed an ACHIM and *Salmonella* coculture assay. As shown in Fig. 1 and Fig. S1 in the supplemental material, different concentrations of ACHIM were mixed with an infectious dose of 10^6^ CFU of *Salmonella* and cocultured for 1, 2, and 5 days anaerobically. The nondiluted ACHIM caused substantial inhibition of *Salmonella* growth. This inhibition was pronounced within the first 24 h and remained strong on day 5. Notably, both ACHIM dilutions displayed significant inhibition after 24 h in a dose-dependent manner (Fig. 1). When the diluted ACHIM established a stable status on day 5, all experimental conditions resulted in a 4-log-unit decrease of *Salmonella* CFU compared to *Salmonella* without ACHIM. These findings suggest that a balanced gut microbiota assists in *Salmonella* clearance.

10.1128/mSystems.00119-18.2 FIG S1 Representative images of control groups of either *Salmonella*-selective agar plates (top and middle rows) or YCFA plates (bottom row) after ACHIM-*Salmonella* coculture assay or phage-bacteria coinfection assay. The treatment conditions are indicated above each plate, and each bacterial streak/column represents one concentration of a serially diluted culture. Download FIG S1, TIF file, 2.1 MB. Copyright © 2018 Hu et al. 2018 Hu et al. This content is distributed under the terms of the Creative Commons Attribution 4.0 International license.

### Both phage and antibiotic treatments clear *Salmonella* in ACHIM.

For severe invasive infections, the routine antimicrobial therapy against Salmonella serovar Typhi and Paratyphi infections is antibiotic treatment. In Sweden, one of the most commonly used antibiotics for invasive *Salmonella* infection is azithromycin. Here we tested the efficacy of phage and azithromycin treatments against *Salmonella* in ACHIM. In order to easily detect microbiota perturbations caused by the treatments, we used a 1:10 dilution of ACHIM. Before conducting the phage-bacterium coinfection assay in ACHIM, *Salmonella* clearance by phage and azithromycin was examined in Luria-Bertani broth (LB). The lowest concentration of either phage cocktail (150 µl of SalmoFresh phage) or antibiotic (250 µl of 10-mg/ml azithromycin) that achieved a complete clearance of 10^6^ CFU *Salmonella* was chosen for the following infection experiments.

**FIG 2:**
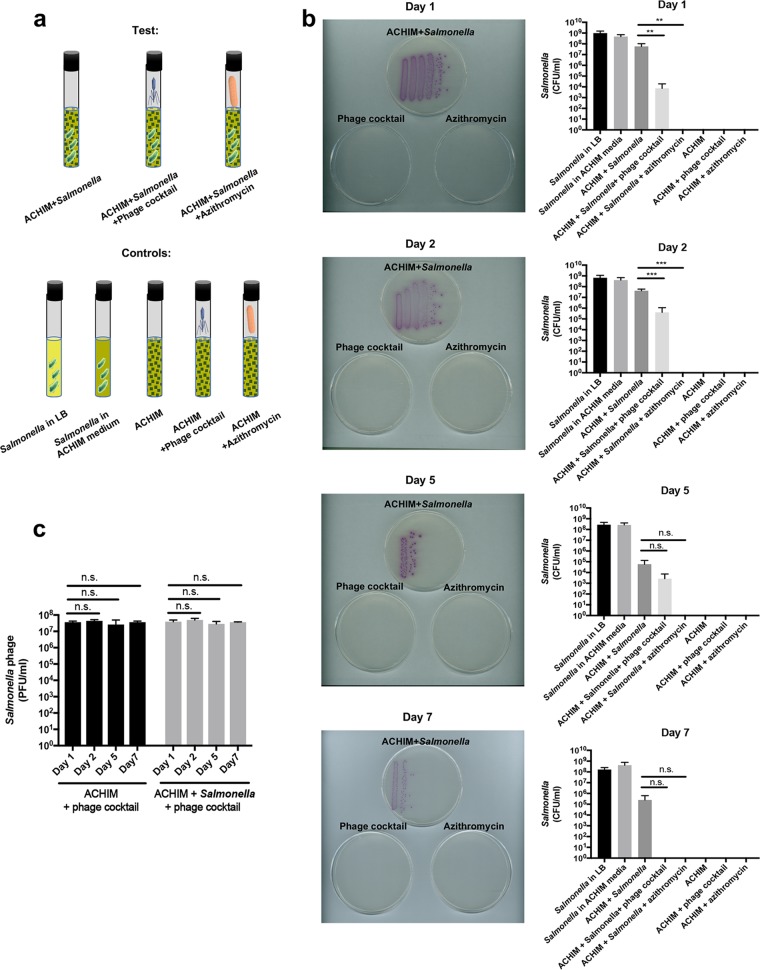
Phage and azithromycin treatments kill *Salmonella*. (a) Schematic of the experimental design. (b) Representative images of *Salmonella* growth on *Salmonella*-selective agar plates and quantification of *Salmonella* (CFU per milliliter) in log scale on days 1, 2, 5, and 7. The treatment conditions are indicated above the plates, and each bacterial streak/column reflects one concentration of a serially diluted culture. (c) Quantification of *Salmonella* phage (PFU/ml) in ACHIM from each experimental day with or without *Salmonella*. Statistical analysis was conducted using one-tailed *t* test analysis. Values that are statistically significantly different for the phage treatment group or azithromycin treatment group and the ACHIM-plus-Salmonella group by one-tailed *t* test analysis are indicated by bars and asterisks as follows: **, *P* < 0.01; ***, *P* < 0.001. Values that are not significantly different are indicated by bars labeled n.s. Values are means plus SD from three experiments.

An infectious dose of *Salmonella* (10^6^ CFU) with either 150 µl of phage cocktail or 250 µl of 10-mg/ml azithromycin was added to ACHIM tubes with corresponding controls under anaerobic conditions. Each tube contained 10 ml culture mix as depicted in Fig. 2a. Strikingly, within 1 day, both of the phage and azithromycin treatments showed a strong reduction of *Salmonella* (Fig. 2b and Fig. S1). This effect lasted for 7 days with the maximum effect on day 7, when both treatments eliminated *Salmonella* (Fig. 2b). Both treatments showed significant reduction of *Salmonella* growth compared to the ACHIM control on days 1 and 2. The decreases on days 5 and 7 for both treatments were dramatic but did not reach statistical significance compared to the control ACHIM with *Salmonella*. This is likely due to the inhibition effect ACHIM has on *Salmonella*, as shown in Fig. 1. Additionally, *Salmonella* growth without ACHIM remained high throughout the experiment (Fig. 2b).

Phages complete their life cycle inside bacteria. In our study, *Salmonella* CFU number decreased significantly after phage treatment. We next sought to identify the phage quantity throughout the experimental setup to determine whether the phage PFU count decreased as the *Salmonella* CFU count dropped. Despite the dramatic decline in *Salmonella* CFU, the phage number stayed stable throughout the experiment regardless of whether the experiment was with *Salmonella* (ACHIM with *Salmonella* and phage) or without *Salmonella* (ACHIM with phage) (Fig. 2c). This suggests that the phage remains in ACHIM independent of *Salmonella* without clearance by the gut microbiota.

### Phage treatment clears *Salmonella* growth with high specificity.

In clinical trials, phage treatment displayed less adverse effects on human gut microbiota compared to antibiotic treatment (23, 27). We investigated the specificity of phage and antibiotic treatments by observing perturbations in ACHIM. Given that there is no optimal approach for counting the total number of bacteria in ACHIM, we used two different methods to present the total ACHIM population after treatments.

**FIG 3:**
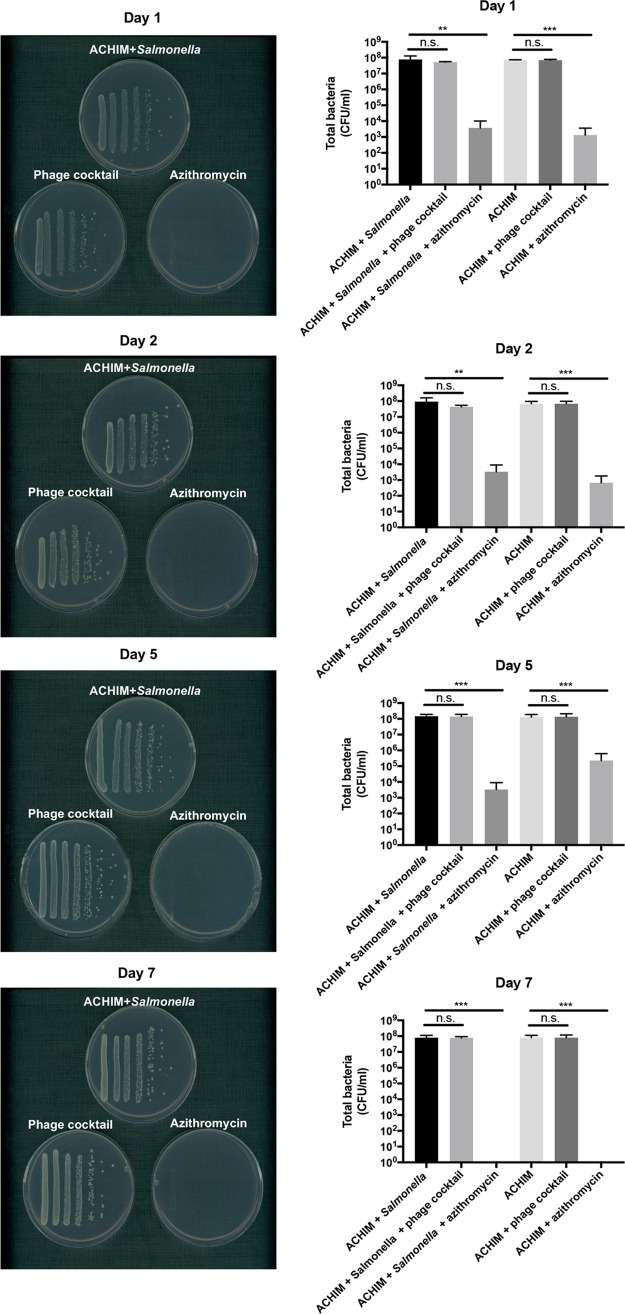
Phage treatment kills *Salmonella* selectively in contrast to azithromycin treatment. (Left) Images of total bacterial growth on YCFA plates imaged on days 1, 2, 5, and 7. The treatment conditions are indicated above each plate, and each bacterial streak/column reflects one concentration of a serially diluted culture. (Right) Total bacterial quantification (CFU per milliliter) for control and treatment conditions in a log scale from each experimental day. Values that are statistically significantly different for the phage treatment group or azithromycin treatment group and the ACHIM-plus-*Salmonella* group by one-tailed T-test analysis are indicated by bars and asterisks as follows: **, *P* < 0.01, ***, *P*  < 0.001. Values that are not significantly different are indicated by bars labeled n.s. Values are means plus SD from three experiments.

YCFA agar plates were recently demonstrated to serve as a foundation for culturing “unculturable” human microbiota (43). Due to this, we used YCFA medium plates to estimate the total bacterial number in ACHIM. As demonstrated in Fig. 3 and Fig. S1, ACHIM with phage treatment displayed a bacterial count similar to those of ACHIM control and ACHIM with *Salmonella*. However, ACHIM CFU decreased dramatically in the antibiotic-treated experimental groups from day 1 and was almost extinguished by day 7 (Fig. 3). These results demonstrate that azithromycin treatment has less specificity than phage treatment and that severe perturbation in ACHIM occurs after only one dose of azithromycin.

**FIG 4:**
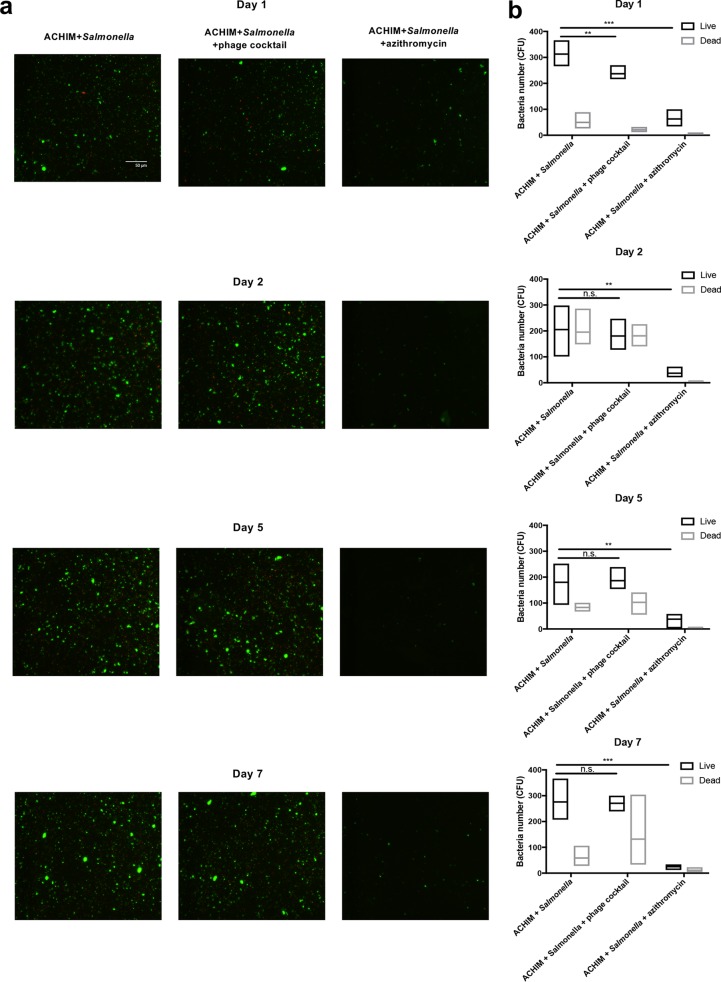
Differential effects on gut bacteria by phage and azithromycin treatments. (a) Representative microscopy images of live (green fluorescent) and dead (red fluorescent) bacteria for phage and antibiotic treatments from each experimental day (days 1, 2, 5, and 7). (b) Quantification of live and dead bacteria for phage and antibiotic treatments imaged on days 1, 2, 5, and 7. The number of live bacteria was quantified in the phage treatment group, the azithromycin treatment group, and the ACHIM-plus-*Salmonella* control group. Values that are statistically significantly different by one-tailed *t* test analysis are indicated by bars and asterisks as follows: **, *P*  < 0.01, ***, *P*  < 0.001. Values that are not significantly different are indicated by bars labeled n.s. Values are means with the maximum and minimum number of the three random fields imaged under the microscope.

Moreover, we also investigated the amount of total bacteria and their viability by staining all bacteria with a LIVE/DEAD staining kit. As seen in Fig. 4a and Fig. S2, not only the total number of bacteria but also the presence of live cells were similar from day 2 in the phage treatment and ACHIM control groups. This supports our data from plate counting. Further, as displayed in Fig. 4b, the proportion of dead bacteria in phage treatment increased from day 1 and reached a maximum on day 2, while shifting toward more live bacteria on day 5 and 7. However, azithromycin treatment killed most of the gut bacteria.

10.1128/mSystems.00119-18.3 FIG S2 Representative microscopy images of live (green fluorescent) and dead (red fluorescent) bacteria for control groups from each experimental day. Download FIG S2, TIF file, 2.6 MB. Copyright © 2018 Hu et al. 2018 Hu et al. This content is distributed under the terms of the Creative Commons Attribution 4.0 International license.

### Phage treatment does not cause perturbations in ACHIM. (i) DNA sequencing.

**FIG 5:**
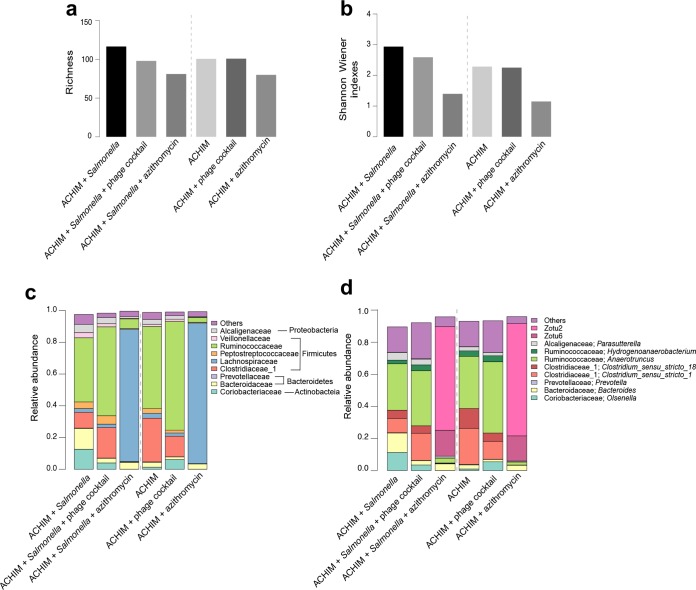
Alpha-diversity indexes and taxonomic composition of the microbiota by DNA-based 16S rRNA sequencing. (a and b) Richness and alpha-diversity in monitored samples. In order to remove bias due to variation in sequence depth, 9,509 reads were subsampled from each sample. The subsampling procedure was repeated 1,000 times, with mean values displayed. The standard deviations (SD) were negligible and are not shown. (c and d) Major taxonomic families and genus in monitored samples. Families and genera containing >1% mean read fractions, are shown, respectively.

To verify whether phage treatment disturbed ACHIM composition, we conducted metabarcoding on the samples from the phage-bacterium coinfection assays. First, we examined the microbial communities of the samples based on their extracted DNA from day 7. The DNA concentrations in all the experimental conditions, including DNA extraction controls and PCR controls from mock bacteria, are listed in Table S1. On average, 23,249 amplicons of the 16S rRNA gene were sequenced from the samples, which results in 196 zero-radius operational taxonomic units (ZOTUs) in total (Table S2). By adding the phage cocktail to ACHIM, compared to ACHIM, no obvious change was observed in both richness (ZOTU counts) and alpha-diversity (Shannon-Wiener index), while the addition of azithromycin resulted in a clear drop in the microbially diverse populations of ACHIM (Fig. 5a and b). In the experimental group, the addition of *Salmonella* increased the microbially diverse populations, while further addition of phage cocktail kept the microbially diverse populations at levels similar to those for ACHIM. Similar to the control group, azithromycin substantially decreased the microbially diverse populations of the gut microbiota (Fig. 5a and b). Here, in order to remove biases caused by the variation in sequencing depth, richness and alpha-diversity indexes were calculated after subsampling to the same number of amplicon reads from each sample. The two indexes displayed high stability in 1,000 iterations of the subsampling procedure, as the maximum standard deviations for the two indexes were only 4.7% and 1.7%.

10.1128/mSystems.00119-18.5 TABLE S1 DNA concentration after extraction or PCR from experimental cultures and controls. Download Table S1, XLSX file, 0.01 MB. Copyright © 2018 Hu et al. 2018 Hu et al. This content is distributed under the terms of the Creative Commons Attribution 4.0 International license.

10.1128/mSystems.00119-18.6 TABLE S2 Data set of ZOTU content from 16S rRNA sequencing. The data set summarizes the number of sequencing reads in ZOTUs of samples from phage and azithromycin treatments. Download Table S2, XLSX file, 0.1 MB. Copyright © 2018 Hu et al. 2018 Hu et al. This content is distributed under the terms of the Creative Commons Attribution 4.0 International license.

DNA sequencing revealed that *Firmicutes* was the most abundant phylum of the samples on day 7, comprising at least 64.9% of the sample reads, and the other major phyla were *Proteobacteria* (notably *Betaproteobacteria*), *Bacteroidetes*, and *Actinobacteria* (Fig. 5c). Congruent with the quantification result from plate enumeration (Fig. 2b), *Salmonella* was basically eliminated from the samples on day 7, with average relative abundance being only 0.02%. The ACHIM microbial community structures were similar after *Salmonella* infection and phage treatment. Compared to the ACHIM control, addition of *Salmonella* and phage treatment resulted in slightly fewer *Clostridia* and more *Bacteroidetes* and *Coriobacteriia*. By depicting the ACHIM microbial community structure at the family level, addition of azithromycin resulted in distinct community profiles with the majority of reads from the *Lachnospiraceae* family that contains two major ZOTUs with only one base difference (ZOTU2 and ZOTU6; Fig. 5d and Fig. S3). Using the SILVA small subunit (SSU) 128 database (44), the database adopted for 16S annotation, the two ZOTUs were unable to be classified at the genus level. A BLAST search in the NCBI nt database revealed ZOTU2 to be identical to the 16S rRNA gene of *Hungatell hathewayi* (from the family *Clostridiaceae*; isolated from human gut microbiota in 2016) and ZOTU6 to be identical to the 16S rRNA gene of an uncultured organism sequenced from human gastrointestinal specimens.

10.1128/mSystems.00119-18.4 FIG S3 ZOTU content in samples from ACHIM and *Salmonella* coculture. All samples were subsampled till the same sequencing level (9,509 reads), and only ZOTUs displaying >0.01% mean read fractions are shown. Download FIG S3, PDF file, 0.1 MB. Copyright © 2018 Hu et al. 2018 Hu et al. This content is distributed under the terms of the Creative Commons Attribution 4.0 International license.

### (ii) RNA sequencing.

**FIG 6:**
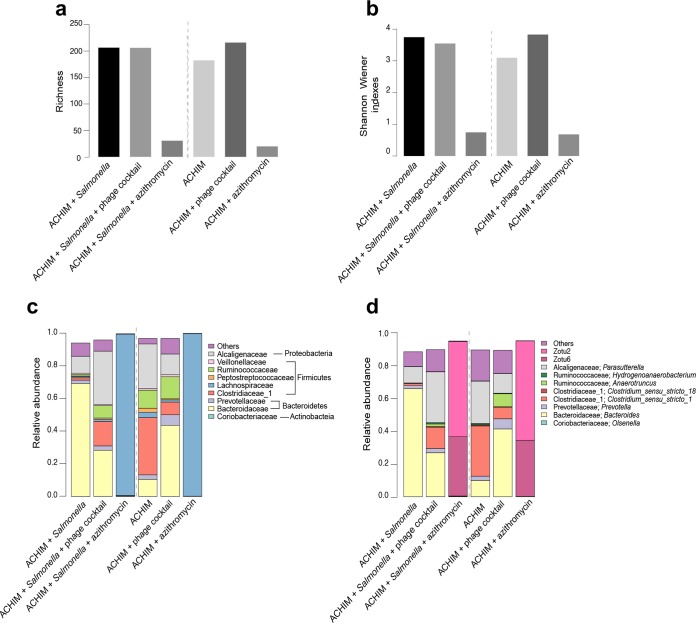
Alpha-diversity indexes and taxonomic composition of the microbiota by RNA-based 16S rRNA sequencing. (a and b) Richness and alpha-diversity in monitored samples. In order to remove bias due to variation in sequence depth, 9,509 reads were subsampled from each sample. The subsampling procedure was repeated 1,000 times, with mean values displayed. The SD were negligible and are not shown. (c and d) Major taxonomic families and genera in monitored samples. Families and genera containing >1% mean read fractions, are shown, respectively.

Since DNA-based sequencing is not able to differentiate live and dead bacteria, we also conducted 16S rRNA sequencing. The RNA concentrations of all the experimental conditions, including extraction controls, are listed in Table S1. On average, 34,666 amplicons of 16S rRNA were sequenced, which resulted in 335 ZOTUs in total (Table S2). A microbial diversity pattern similar to that of the DNA sequencing results was detected. Samples with antibiotic treatment displayed the lowest diversity among all conditions, and the diversity indexes of samples with and without antibiotics were substantially different. A fivefold difference was observed in the Shannon index for the phage-treated samples compared to the antibiotic-treated samples (Fig. 6a and b). Similar to the DNA sequencing results, the samples with azithromycin mainly contained ZOTU2 and ZOTU6, while the other samples were composed of common phyla from the human gut microbiota (Fig. 6c and d and Fig. S3). Notably, both ZOTU2 and ZOTU6 can be found in ACHIM but at a very low number of reads. In contrast, ZOTU2 and ZOTU6 were present at very high numbers of reads after antibiotic treatment (Fig. 5 and 6). This indicates that bacteria containing ZOTU2 and ZOTU6 are likely to be antibiotic resistant. Furthermore, the compositions of ZOTUs from both DNA sequencing and RNA sequencing further explicitly demonstrate the taxa activities (Fig. S3).

### (iii) PCoA and clustering.

**FIG 7:**
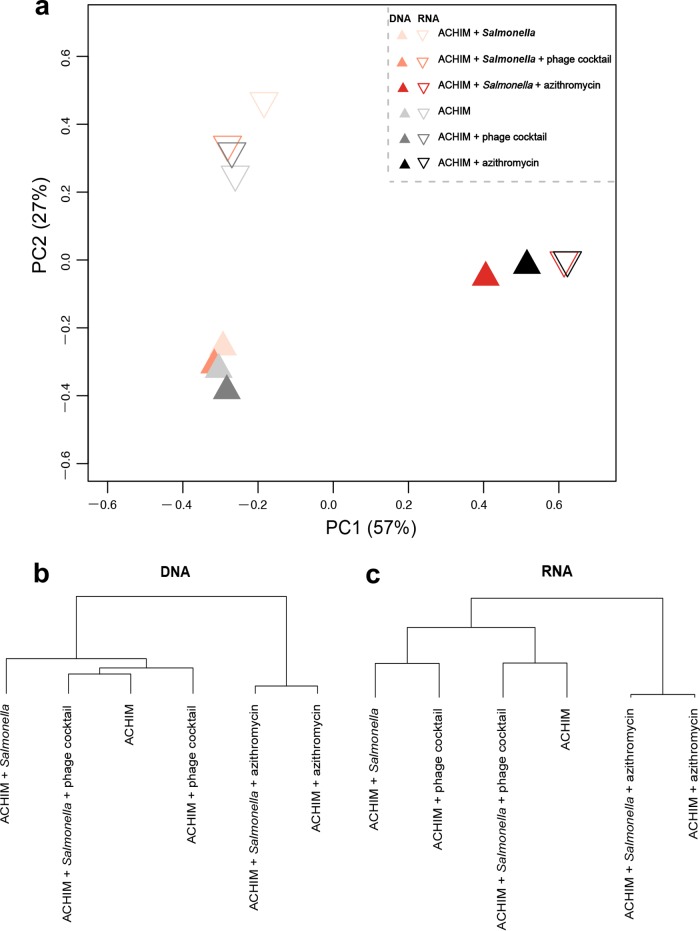
Principal-coordinate analysis (PCoA) and hierarchical clustering based on beta-diversity calculated using Bray-Curtis matrix. (a) Both DNA- and RNA-based sequences were combined to calculate beta-diversity. (b and c) DNA- and RNA-based sequences were used separately to calculate the beta-diversity of microbial content in samples.

To demonstrate the degree of dissimilarity of the ACHIM microbial communities across all treatments, we employed principal-coordinate analysis (PCoA) and hierarchical clustering to visualize the variations in beta-diversity (based on Bray-Curtis matrix) (Fig. 7). In PCoA analysis, the addition of antibiotics explained the largest fraction of the variation in the bacterial communities of all samples (57%; Fig. 7a). The sequencing materials (DNA- or RNA-based sequencing) resulted in a separation of the microbial community profiles. However, phage treatment profiles were always grouped together with ACHIM controls, while antibiotic treatment had strong effects on microbial community structure. In addition, the clustering results further elucidated the similarities of samples in terms of their microbial content. As we expected, in contrast to antibiotic treatment, phage treatment maintained the microbiota of ACHIM after *Salmonella* infection (Fig. 7b and c).

## DISCUSSION

The gut microbiota plays an important role in providing protection against enteric pathogens through various mechanisms (45, 46). We demonstrate that, to a certain extent, ACHIM inhibits *Salmonella* growth. This mechanism is possibly due to colonization resistance either by the bacteriocins or effectors secreted by bacteria in ACHIM, or by direct bacterial interaction such as the type VI secretion system (T6SS). However, the exact mechanisms by which and how the bacteria regulate the clearance of pathogen infection remain to be discovered. By keeping pathogens restrained, the gut microbiota assists the immune system by clearing pathogenic infections (47). This emphasizes the importance of a balanced gut microbiota and might partly explain why most *Salmonella* infections are cured without treatment.

Furthermore, ACHIM, a proxy for the human gut microbiota, provides an attractive biological culture for studying interactions between specific bacterial strains and for comparing various therapeutic treatments. ACHIM can be screened in a high-throughput manner with easily interpreted results and is superior to mouse models since numerous human bacteria are unable to colonize the mouse gut. Fecal microbial transplants have proven effective in treating several diseases, most notably Clostridium difficile diarrhea (48). ACHIM has been used for fecal transplant with promising results, notably without the need to find fecal donors or screen for pathogens (17, 49–51). With its potential as an alternative treatment for other enteric infections, ACHIM could potentially be used as the standard balanced gut microbiota culture for fecal microbiota transplants.

In contrast to phage treatment, antibiotic treatment efficiently but indiscriminately ablated virtually all ACHIM bacteria. The indiscriminate effect of azithromycin, including targeting of anaerobic commensal bacteria, leads to dysbiosis and suppression of colonization resistance, which consequently results in relapse of, and susceptibility to, other infections (52, 53). Azithromycin fundamentally decreased gut microbiota volume and diversity, emphasizing the destructive power of broad-spectrum antibiotics. Sequencing data confirmed that phage treatment largely maintains the gut microbiota composition, which proves the specificity and reliability of phage treatment for bacterial infections. Such adverse effects and the threat of multidrug-resistant bacteria highlight the importance of phage treatment as an alternative approach to combat bacterial infections.

The taxonomic compositions in the RNA-based analysis are different from the taxonomic compositions in the DNA-based analysis. This is largely due to the inability of discriminating live and dead bacteria with DNA-based analysis. Furthermore, the transcriptome of certain bacteria may also affect taxonomic compositions. In addition, our study utilizes an *in vitro* microbiota culture without considering host cell responses. However, this provides us a clear-cut culture for studying whole-gut microbiota-pathogen interactions for up to 7 days. Studies beyond 7 days are not feasible using this culture, as back-dilutions of the bacteria would enforce a reset of the experiments.

In summary, ACHIM provides a feasible platform for investigating the effects of various treatments on the gut microbiota. ACHIM naturally inhibits *Salmonella* infection. Phage treatment is an efficient and selective approach for eradicating pathogenic infections in the gut microbiota. However, the exact mechanisms by which the gut microbiota clears pathogenic infections and synergizes with phages remain unknown. In contrast to the detrimental effects of antibiotic treatment on commensal bacteria in the gut microbiota, phage treatment leaves the microbiota balance virtually without perturbation.

## MATERIALS AND METHODS

### Bacterial strains and culture conditions.

The *Salmonella* strain used in this study was Salmonella enterica serovar Enteritidis PT4, kindly provided by Derek Pickard and Gordon Dougan at the Wellcome Trust Sanger Institute, Hinxton, Cambridge, United Kingdom, to Å. Sjöling. *Salmonella* was cultured at 37°C with aeration in Luria-Bertani broth (LB) (Sigma-Aldrich, Germany). Many *Salmonella* food-borne outbreaks are caused by an infectious dose of ≤10^3^ *Salmonella* cells, but higher doses are associated with high rates of attack and short periods of incubation (54). Therefore, a dose of 10^6^ CFU was used during our experiments (10^5^ CFU/ml in 10-ml experiment volume).

Anaerobically cultivated human intestinal microflora (ACHIM) was originally obtained from fresh feces of a healthy Scandinavian donor on an ordinary Western diet. The culturing was conducted as described previously (17) (patent application WO 2013/053836A). The feces/culture were investigated for the absence of common viral infections, including hepatitis A, B, and C virus, cytomegalovirus, Epstein-Barr virus, human immunodeficiency virus (HIV), rotavirus, and calicivirus, as well as common bacterial infections, including *Salmonella*, *Shigella*, *Campylobacter*, *Yersinia*, and C. difficile. This culture was recultivated every week under anaerobic conditions with resazurin as an indicator. The 1-week culture would typically contain approximately 10^9^ CFU.

### Phage and antibiotic treatments.

The phage treatment SalmoFresh was kindly provided by Intralytix (MD, USA) and is the subject of U.S. patents 7,674,467 and 8,685,696. This phage cocktail contains a mix of six strictly lytic phages that selectively kill *Salmonella.* The preparation is listed as GRAS (generally recognized as safe) by the U.S. Food and Drug Administration (FDA) for direct application onto poultry, fish, shellfish, and fresh and processed fruits and vegetables (GRAS notice [GRN] 435). SalmoFresh was stored at 4°C protected from light. The experiments were carried out within a month after opening a new SalmoFresh bottle. The PFU from the bottle used in our study was tested to be at the concentration of ca. 0.9 × 10^9^ PFU/ml. Azithromycin (Sigma-Aldrich, Germany) was chosen as the antibiotic, as it is increasingly used for the treatment of invasive *Salmonella* infections (55).

### *Salmonella* enumeration.

This experiment aimed to evaluate the total CFU of *Salmonella* in the test tubes. *Salmonella-*selective agar plates, which selectively isolate *Salmonella* species from other bacteria, were made from CHROMagar *Salmonella* plus base (Thermo Fisher Scientific, MA, USA) according to the manufacturer’s suggested protocol. Cultured bacteria were diluted in 96-well plates, and 10-µl aliquots from each dilution were plated on *Salmonella*-selective agar plates using a multichannel pipette. The agar plates were positioned at a ca. 30 to 45° angle, so that bacterial samples formed a streak/column on the agar plates. The plates were incubated at 37°C with aeration overnight. The next day, *Salmonella* bacteria were counted in the range of 30 to 300 CFU dilutions. *Salmonella* (CFU/ml) was then quantified according to the dilution factors.

### ACHIM and *Salmonella* coculture assay.

The effects of ACHIM on *Salmonella* growth were investigated with this assay. One-week ACHIM (10^9^ CFU) and different ACHIM concentrations, including 1:10 (10^8^ CFU) and 1:100 dilutions (10^7^ CFU) with ACHIM medium were inoculated together with the overnight culture of *Salmonella* (10^6^ CFU). ACHIM medium without ACHIM bacteria and ACHIM back dilution (10^8^ CFU) without *Salmonella* were included as controls. Inoculation day was considered day 0. All tubes were grown anaerobically in a 10-ml culture standard. On days 1, 2, and 5, *Salmonella* was counted by serial dilution and plated on selective plates as described above.

### Phage-bacterium coinfection assay.

This experiment aimed to compare the efficiency of both phage and azithromycin treatment against *Salmonella* growth in ACHIM. *Salmonella* (10^6^ CFU) and fresh back diluted ACHIM (10^8^ CFU) were cocultured in ACHIM medium at 10-ml volume. In addition, *Salmonella* phage cocktail or azithromycin was added to the bacteria mix. ACHIM without *Salmonella* and ACHIM without *Salmonella* but with phage cocktail or azithromycin were cultured at the same time as controls. All tubes were grown anaerobically and monitored on days 1, 2, 5, and 7. On each day, *Salmonella* CFU and total bacteria number were counted as described above.

Since there are no plates available that can serve as a growth foundation for all gut bacteria, we used modified YCFA medium plates, recommended by Deutsche Sammlung von Mikroorganismen und Zellkulturen (DSMZ), supplemented with 2 g/liter maltose and 2 g/liter cellobiose, to estimate the total bacterial number (all reagents from Sigma-Aldrich, Germany) (43). YCFA plates were grown anaerobically at 37°C. The next day, bacterial numbers in the range of 30 to 300 CFU were counted. Total bacterial numbers from each condition were determined by dilution factors and then compared from each experimental day.

### Phage enumeration.

*Salmonella* phage PFU count was determined by phage plaque assay. Briefly, 500 µl of culture mixed from tubes with phage treatment on each experimental day was filtered through a 0.2-µm filter. Filtrate was added to 5-ml culture tubes with soft LB agar (LB broth plus 9-g/liter agar) (Sigma-Aldrich, Germany), 200 µl overnight *Salmonella* culture, and 100 µl of the serial diluted supernatant. The mixture was poured into LB plates and left until top agar hardened. LB plates were incubated at 37°C overnight. Each plaque, which indicates an initial infection followed by lysis of neighboring bacteria, was quantitated by the number of PFU per milliliter.

### LIVE/DEAD bacterial staining.

Both live and dead bacteria were determined by staining with LIVE/DEAD BacLight bacterial viability dyes (Thermo Fisher Scientific, MA, USA). Bacterial cultures on days 1, 2, 5, and 7 were obtained and filtered through a 40-µm filter (Sigma-Aldrich, Germany). After spinning at 13,000 rpm for 3 min, pellets were washed once with 0.9% (wt/vol) sodium chloride solution. Bacterial viability dyes were mixed according to the protocol and added at a 1:1,000 concentration. After a light-protected incubation at room temperature for 30 min, 10 µl bacteria from each condition were mixed with 10 µl of 1% UltraPure liquid agarose (Thermo Fisher Scientific, MA, USA) at 45°C. Then 10 µl of the mixture was dropped on a glass slide and fixed by a cover slide before the mix became solid. Slides were imaged on an Axiovert 200M inverted microscope (Carl Zeiss, Germany), using 20× magnification objective lenses and 4-s exposure time.

### Genetic material extraction and 16S rRNA gene sequencing.

Sequencing was used to highlight how different treatments disturbed ACHIM composition. Total DNA and RNA were extracted separately from 1-ml portions of the final cultures (the cultures on day 7) by using DNeasy Blood & Tissue kit (Qiagen, Germany) and Qiagen RNeasy minikit (Qiagen, Germany) according to the manufacturer’s protocols. Before DNA extraction, all samples were treated by adding 50 µl water, boiled at 100°C for 15 min, followed by addition of 180 µl lysis buffer (ATL buffer from DNeasy Blood & Tissue kit), and incubated at room temperature overnight. Before RNA extraction, all RNA samples were treated with RNAprotect bacterial reagent (Qiagen, Germany) according to the protocol. To enable amplicon sequencing, the extracted RNA was subsequently converted to cDNA using QuantiTect reverse transcription kit (Qiagen, Germany). A pair of published primers 341F (F stands for forward) (CCTACGGGNGGCWGCAG) and 805R (R stands for reverse) (GACTACHVGGGTATCTAATCC) were used for targeting the V3-V4 regions of the bacterial 16S rRNA gene (56). The genomic DNA of a mock bacterial community supplied by BEI Resources (catalog no. HM-782D: Manassas, USA) and DNA-free water were used as the positive and negative controls for PCR performance, respectively. Sequencing was performed on an Illumina MiSeq platform (Illumina, CA, USA) at Clinical Genomics/SciLifeLab Stockholm.

### Sequence processing.

The operational taxonomic unit (OTU) table used in this study was constructed following steps from available workflows using Cutadapt, Usearch, and Vsearch for quality trimming, OTU picking, and taxonomic annotation (57–60). Parameters used in the key steps were as follows: when merging paired-end reads that passed preliminary quality trimming (minimum quality value of 15; minimum length of 120 bp, maximum 3 N bases), we discarded the read pairs that can result in merged reads with the number of expected errors of >3 or not in the size range of 380 bp ∼520 bp to further improve the quality of amplicon sequences. Usearch denoising function (unoise3) was applied to generate zero-radius OTUs (ZOTUs), which were classified by using SILVA 128 small subunit (SSU) database. In addition to the SILVA database, manual annotation by conducting BLAST searches in the NCBI nt database was performed on the ZOTUs of interest (61). The merged reads were mapped back to OTUs with higher than 0.98 similarities. A table of ZOTU counts per sample combined with their taxonomic information was generated for the downstream analysis (see Table S2 in the supplemental material).

### Data analysis.

We employed the programming language R to conduct the analysis and plotting for sequencing data. The R codes coupled with their generated plots were recorded in a PDF by using Jupyter Notebook (see Text S1 in the supplemental material). Briefly, to demonstrate the bacterial community composition at different taxonomic levels, we summed up the normalized read counts for each taxonomy and then applied R package RColorBrewer to demonstrate the taxonomic content in stacked barplots for each sample (62). The microbiome content of each sample at ZOTU level is depicted in a heatmap by using the R package Pheatmap (63). In order to calculate sample richness and alpha-diversity fairly, a subsampling procedure with 1,000 times iteration was conducted by using the rrarefy function from R package vegan (64). With the functions offered in the same package, alpha-diversity (Shannon-Wiener index) for each sample and beta-diversity (Bray-Curtis distance) among samples were calculated. Based on the generated beta-diversity dissimilarity matrix, hierarchical clustering and PCoA analysis were conducted by using R packages cluster and ade4 to demonstrate the differences among the microbiome of each sample (65).

10.1128/mSystems.00119-18.1 TEXT S1 R script used to generate all the sequencing analysis results in this study. Download Text S1, PDF file, 1.1 MB. Copyright © 2018 Hu et al. 2018 Hu et al. This content is distributed under the terms of the Creative Commons Attribution 4.0 International license.

### Ethics statement.

ACHIM was originally obtained from fresh feces of a healthy Scandinavian donor anonymously. This study has received ethical permission from the Ethical Committee at Karolinska Institutet, Stockholm, Sweden.

### Data availability and material.

All data generated or analyzed during this study are included in the published article (and its supplemental material files).

### Accession number(s).

The sequencing reads have been submitted to the European Nucleotide Archive (ENA) under accession number PRJEB24795.
