# Supplementary figures and images for: Bacteriophages Synergize with the Gut Microbial Community To Combat Salmonella
Source: mSystems. 2018 Oct 2;3(5):e00119-18. doi: 10.1128/mSystems.00119-18 (PMC6172775; doi:10.1128/mSystems.00119-18)

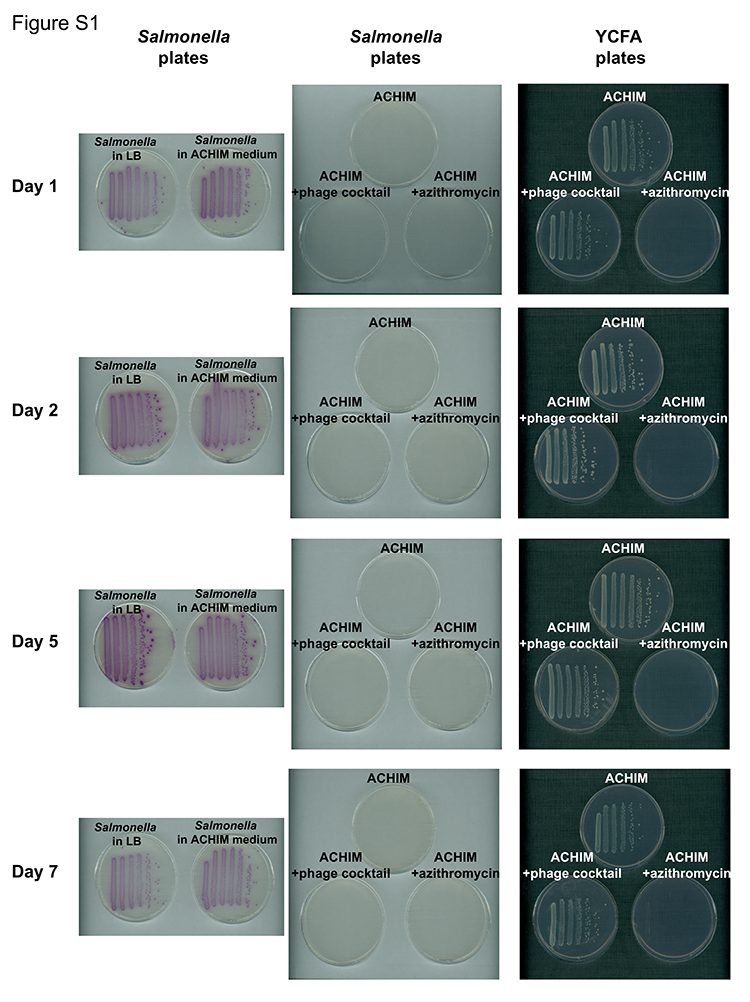

Supplement: FIG S1 [file sys005182267sf1.tif]

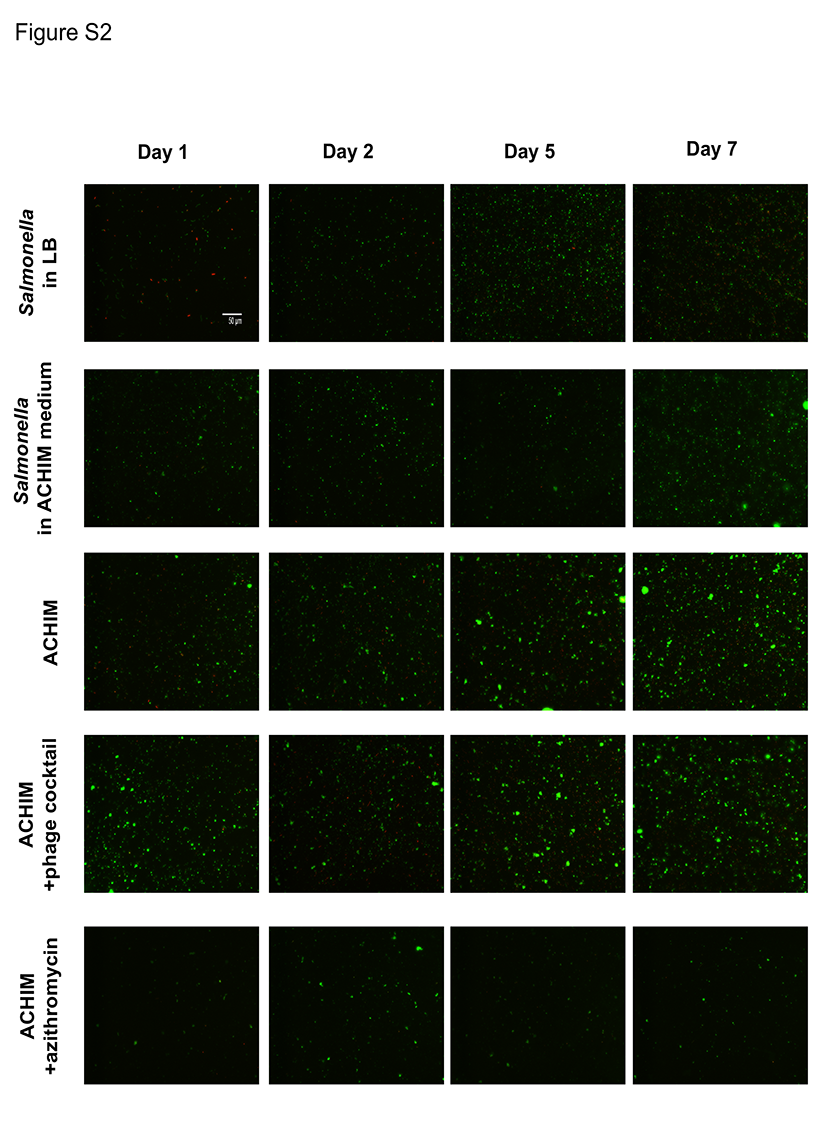

Supplement: FIG S2 [file sys005182267sf2.tif]

Figure S3

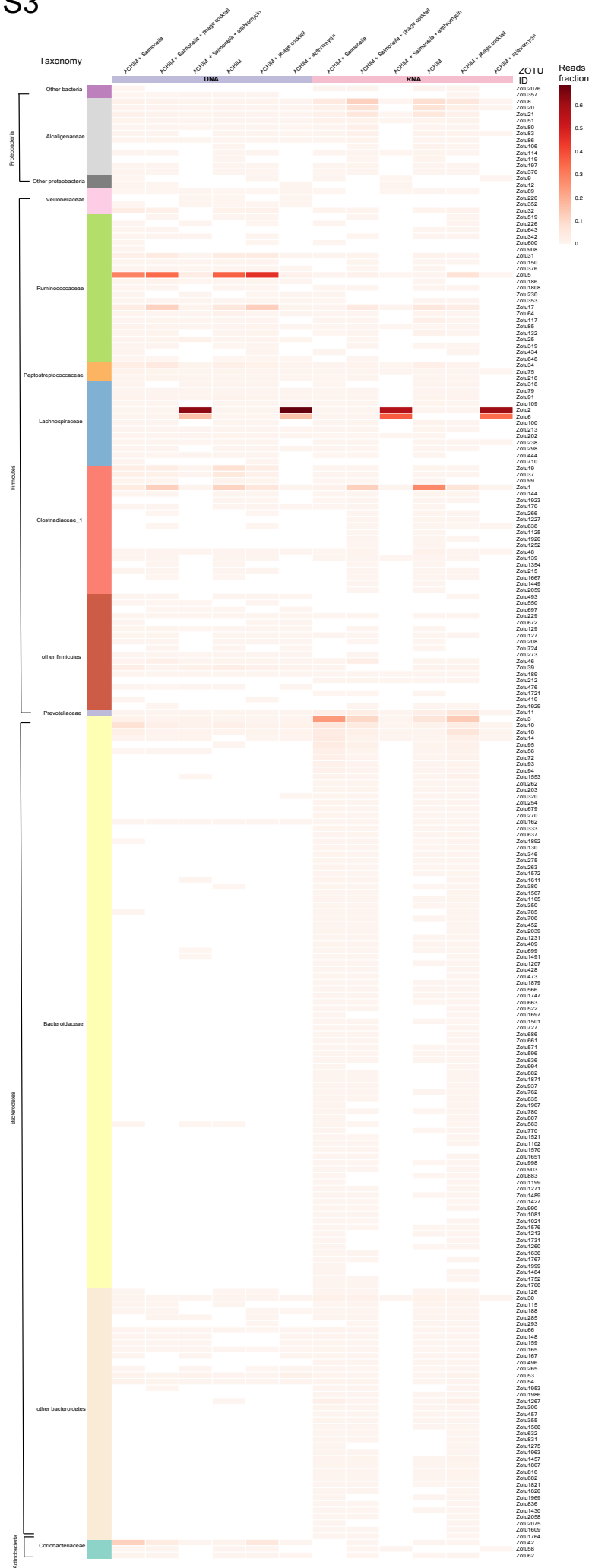

Supplement: FIG S3 [file sys005182267sf3.pdf]
