# Supplementary material for: Bacteriophages Synergize with the Gut Microbial Community To Combat Salmonella
Source: mSystems. 2018 Oct 2;3(5):e00119-18. doi: 10.1128/mSystems.00119-18 (PMC6172775; doi:10.1128/mSystems.00119-18)
Supplement: TEXT S1 [file sys005182267s1.pdf]

In [1]:
```
# Data analysis for the manuscript:
#   Bacteriophages synergize with the gut microbial community to combat Sa
lmonella (mSystems)
# R Script version: 3.0
# Date: 2018-08-07
# Author: yue.hu@ki.se
```

In [2]:
```
# install packages
install.packages(c("vegan","cluster", "ape", "pheatmap", "RColorBrewer"))
lapply(list("vegan","cluster", "ape", "pheatmap", "RColorBrewer"), require
, character.only = TRUE)
```

```
Updating HTML index of packages in '.Library'
Making 'packages.html' ... done
Loading required package: vegan
Loading required package: permute
Loading required package: lattice
This is vegan 2.4-5
Loading required package: cluster
Loading required package: ape
Loading required package: pheatmap
Loading required package: RColorBrewer
```

1. TRUE
2. TRUE
3. TRUE
4. TRUE
5. TRUE

In [3]:
```
## read DNA concentration table
path = "~/171013_ACHIM_salmonella_phage/mSystems/Supplementary_tables/"
file1 = paste0(path,"Supplementary_table_1.tsv") # S1 table: DNA concentra
tion after extraction or PCR
conc_tab <- read.delim(file1,sep = "\t")
sample <- as.matrix(conc_tab$sample_id)

group_dna <- grep("^DNA",sample)
group_rna <- grep("^cDNA",sample)
group_ctrl <- grep("Control", sample)
```

In [4]:
```
## read OTU table
file2 <- paste0(path,"Supplementary_table_2.tsv") # S2 table: OTU table
OTU_tab <- read.delim(file2,sep = "\t")
str(OTU_tab)

taxa <- as.matrix(OTU_tab$taxonomies)
seqs <- as.matrix(OTU_tab$sequences)
seq_id <- as.matrix(OTU_tab$seq_id)
seq_id<- sapply(seq_id,paste0,"_")

reads_16S <- as.matrix(OTU_tab[2:(ncol(OTU_tab)-2)])
```

```
'data.frame':	401 obs. of  17 variables:
 $ seq_id                            : Factor w/ 401 levels "Zotu1","Zotu
10",..: 191 247 109 317 110 307 300 262 208 74 ...
 $ DNA_ACHIM_Salmonella              : int  1 10 0 0 0 96 6 3549 5 0 ...
 $ DNA_ACHIM_Salmonella_phage        : int  0 6 0 0 0 48 9 939 2 0 ...
 $ DNA_ACHIM_Salmonella_azithromycin : int  0 3 0 0 0 0 0 5 2 0 ...
 $ DNA_ACHIM                         : int  0 19 0 0 0 17 2 131 0 0 ...
 $ DNA_ACHIM_phage                   : int  0 17 0 0 0 34 7 1412 2 0 ...
 $ DNA_ACHIM_azithromycin            : int  0 9 0 0 0 6 1 19 4 0 ...
 $ cDNA_ACHIM_Salmonella             : int  9 0 0 0 0 0 0 2 0 197 ...
 $ cDNA_ACHIM_Salmonella_phage       : int  22 1 0 1 0 3 1 11 1 103 ...
 $ cDNA_ACHIM_Salmonella_azithromycin: int  0 0 0 0 0 0 42 0 0 0 ...
 $ cDNA_ACHIM                        : int  18 0 0 0 0 1 1 10 0 83 ...
 $ cDNA_ACHIM_phage                  : int  31 2 4 0 0 2 0 5 0 388 ...
 $ cDNA_ACHIM_azithromycin           : int  0 1 0 0 0 0 9 0 0 1 ...
 $ Control_PCR_pos                   : int  0 0 0 0 1 0 0 0 0 0 ...
 $ Control_PCR_neg                   : int  0 0 0 0 0 0 0 0 0 0 ...
 $ taxonomies                        : Factor w/ 90 levels "Bacteria;","B
acteria;Actinobacteria;Actinobacteria;Actinomycetales;Actinomycetaceae;Ac
tinomyces;",..: 1 1 1 1 2 3 4 5 5 6 ...
 $ sequences                         : Factor w/ 401 levels "TAGGGAATATTG
CTCAATGGGGGAAACCCTGAAGCAGCAACGCCGCGTGGAGGATGAAGGTTTTAGGATTGTAAACTCCTTTTGT
TAGAGAAGATAATGACGGTATCTAAC"| __truncated__,..: 220 201 363 279 191 362 36
4 359 361 168 ...
```

In [5]:
```
# reads normalization till OTU level
norm_reads_16S <- reads_16S
for (i in 1:ncol(reads_16S)) {
  norm_reads_16S[,i] <- reads_16S[,i]/sum(reads_16S[,i])
}
```

In [6]:
```
# normalization at different taxonomic level  (group Proteobacteria togeth
er)
taxonomy <- taxa
reads<- reads_16S

taxalevel <- c("domain","phylum","class","order","family","genus","specie
s")
for (level in 2:7){
  clades <- levels(factor(taxonomy))
  for (i in 1:length(clades)) {
    if (length( matrix(unlist(strsplit(clades[i],";")), 1)[1,] ) >= level)
 {
      string <- matrix(unlist(strsplit(clades[i],";")), 1)[1,1]
      for (j in 2:level) {
        string <- paste(string, matrix(unlist(strsplit(clades[i],";")), 1)
[1,j], sep = ";")
      }
      clades[i] <- string
    } else {
      clades[i] <- NA
    }
  }
  clades <- levels(factor(clades))
  clades <- clades[ setdiff(c(1:length(clades)), grep("no_match", clades))
 ]
  clades[length(clades) + 1] <- "others"
  clade_reads <- matrix(nrow = length(clades), ncol = ncol(reads))
  colnames(clade_reads) <- colnames(reads)
  rownames(clade_reads) <- clades
  all_these <- vector(length = 0)
  for (i in 1:length(clades)) {
    these <- grep (clades[i], taxonomy)
    all_these <- union(these, all_these)
    for (j in 1:ncol(clade_reads)) {
      clade_reads[i,j] <- sum(reads[these,j])
    }
  }
  others <- setdiff(c(1:length(taxonomy)), all_these)
  for (j in 1:ncol(clade_reads)) {
    clade_reads[length(clades),j] <- sum(reads[others,j])
  }
  norm_clade_reads <- clade_reads
  for (i in 1:ncol(clade_reads)) {
    norm_clade_reads[,i] <- clade_reads[,i]/sum(clade_reads[,i])
  }
  assign(paste0(taxalevel[level],"_reads"), clade_reads)
  assign(paste0("norm_",taxalevel[level],"_reads"), norm_clade_reads)
  assign(taxalevel[level],clades)
}
```

In [7]:

```
############################################################
# community composition

# family level

# DNA & RNA
group <- c(group_dna,group_rna)
n <- 0.01 # cut-off: only demonstrate the families with average relative a
bundance >1%
matr <- norm_family_reads[,group] #choose the norm taxa you want to use
ok <- which(apply(matr, 1, mean) >= n)
matr <- matr[ok,]
rownames(matr) = sapply(strsplit(rownames(matr), ";"), "[", 5)
rownames(matr)[nrow(matr)] <- "Others"
color = brewer.pal(nrow(matr),"Set3")
label = c("ACHIM + Salmonella", "ACHIM + Salmonella + phage cocktail", "AC
HIM + Salmonella + azithromycin",
         "ACHIM", "ACHIM + phage cocktail", "ACHIM + azithromycin")


options(repr.plot.width=14, repr.plot.height=10)
par(oma = c(2,0,0,0), mar = c(18,7,3,1))


# DNA

barplot(matr[,group_dna] ,col = color, legend = T, las =2,cex.names = 0.7,
ylim = c(0,1),xlim = c(0,15),
        main = "DNA community composition (family level; Fig 5c)",cex.main
 = 2,
        args.legend = list(x = "topright",bty="n",cex=0.8,x=16),xaxt="n")
text((seq_along(sample[group_dna])-0.2)*1.2, -0.03,
     srt = 45, adj= 1, xpd = TRUE,
     labels = label, cex=1, font = 2)
abline(v=3.67, col="grey", lty=2, lwd=3)

# RNA

barplot(matr[,group_rna] ,col=color, legend=T, las =2,cex.names = 0.7,ylim
 = c(0,1),xlim = c(0,15),
        main = "RNA community composition (family level; Fig 6c)",cex.main
 = 2,
        args.legend = list(x="topright",bty="n",cex=0.8,x=16),xaxt="n")
text((seq_along(sample[group_rna])-0.2)*1.2, -0.03,
     srt = 45, adj= 1, xpd = TRUE,
     labels = label, cex=1, font = 2)
abline(v=3.67, col="grey", lty=2, lwd=3)
```

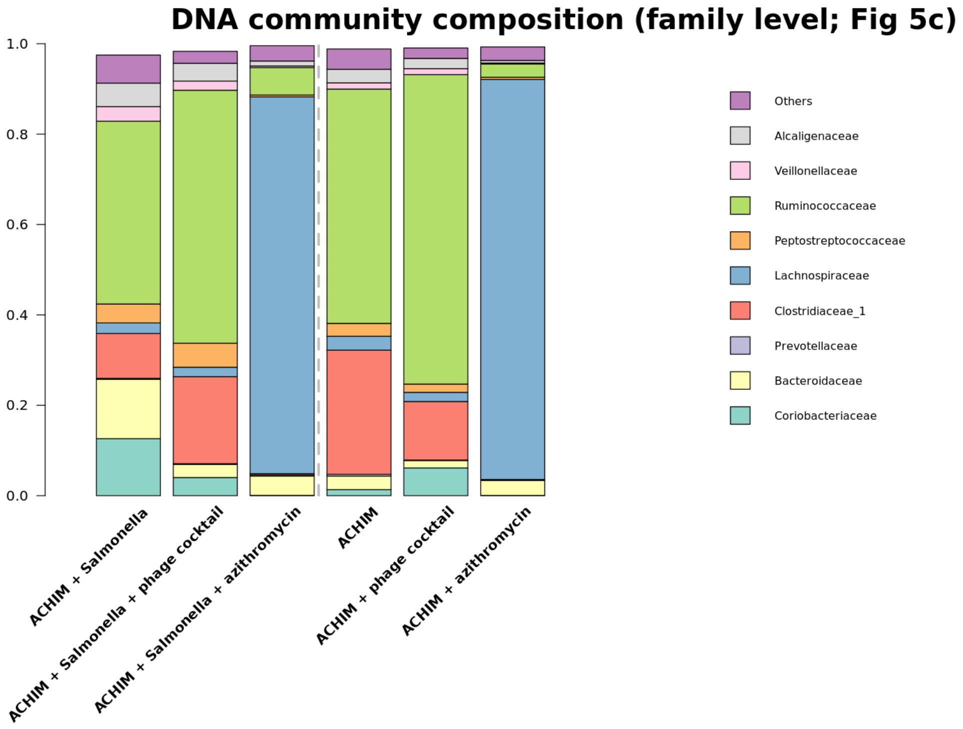
DNA community composition (family level; Fig 5c)
1.0
0.8
0.6
0.4
0.2
0.0
ACHIM + Salmonella
ACHIM + Salmonella + phage cocktail
ACHIM + Salmonella + azithromycin
ACHIM
ACHIM + phage cocktail
ACHIM + azithromycin
Others
Alcaligenaceae
Veillonellaceae
Ruminococcaceae
Peptostreptococcaceae
Lachnospiraceae
Clostridiaceae_1
Prevotellaceae
Bacteroidaceae
Coriobacteriaceae

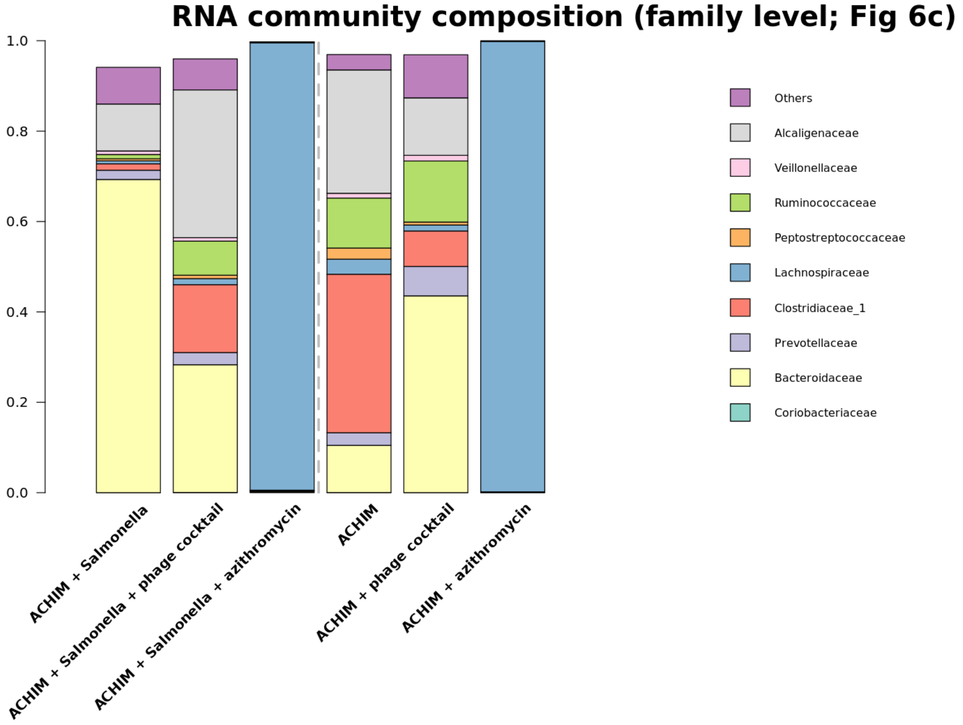
RNA community composition (family level; Fig 6c)
1.0
0.8
0.6
0.4
0.2
0.0
ACHIM + Salmonella
ACHIM + Salmonella + phage cocktail
ACHIM + Salmonella + azithromycin
ACHIM
ACHIM + phage cocktail
ACHIM + azithromycin
Others
Alcaligenaceae
Veillonellaceae
Ruminococcaceae
Peptostreptococcaceae
Lachnospiraceae
Clostridiaceae_1
Prevotellaceae
Bacteroidaceae
Coriobacteriaceae

In [8]:

```
# genus level

matr <- norm_genus_reads[,group]
n <- 0.01 # cut-off: only demonstrate the genera with average relative abu
ndance >1%
ok <- which(apply(matr, 1, mean) >= n)
matr <- matr[ok,]
rownames(matr) = paste0(sapply(strsplit(rownames(matr), ";"), "[", 5),";",
sapply(strsplit(rownames(matr), ";"), "[", 6))
rownames(matr)[nrow(matr)] <- "Others"

# extract Zotu2 and Zotu6 (the two abundant OTU from "Lachnospiraceae")
matr_seq_6_2 <- norm_reads_16S[match(c("Zotu6_","Zotu2_"),seq_id),group]
rownames(matr_seq_6_2) <- c("Zotu6","Zotu2")
# add matr_seq_6_2 to the plot matrix
matr <- rbind(matr[1:(nrow(matr)-1),],matr_seq_6_2,matr[nrow(matr),]-colSu
ms(matr_seq_6_2))
rownames(matr)[nrow(matr)] <- "Others"

color <- c("#8DD3C7", "#FFFFB3", "#BEBADA","#FB8072" ,"indianred3","#B3DE6
9","seagreen4",
           "#D9D9D9" , "hotpink3","hotpink1" ,"#BC80BD")



options(repr.plot.width=14, repr.plot.height=10)
par(oma = c(2,0,0,0), mar = c(18,7,3,1))


# DNA
barplot(matr[,group_dna] ,col=color, legend=T, las =2,cex.names = 0.7,ylim
 = c(0,1),xlim = c(0,15),
         main = "DNA community composition (genus level; Fig 5d)",cex.main
 = 2,
        args.legend = list(x="topright",bty="n",cex=0.8,x=20), xaxt="n")

text((seq_along(sample[group_dna])-0.2)*1.2, -0.03,
     srt = 45, adj= 1, xpd = TRUE,
     labels = label, cex=1, font = 2)
abline(v=3.65, col="grey", lty=2, lwd=3)

#RNA
barplot(matr[,group_rna] ,col=color, legend=T, las =2,cex.names = 0.7,ylim
 = c(0,1),xlim = c(0,15),
         main = "RNA community composition (genus level; Fig 6d)",cex.main
 = 2,
        args.legend = list(x="topright",bty="n",cex=0.8,x=20), xaxt="n")
text((seq_along(sample[group_rna])-0.2)*1.2, -0.03,
     srt = 45, adj= 1, xpd = TRUE,
     labels = label, cex=1, font = 2)
abline(v=3.65, col="grey", lty=2, lwd=3)
```

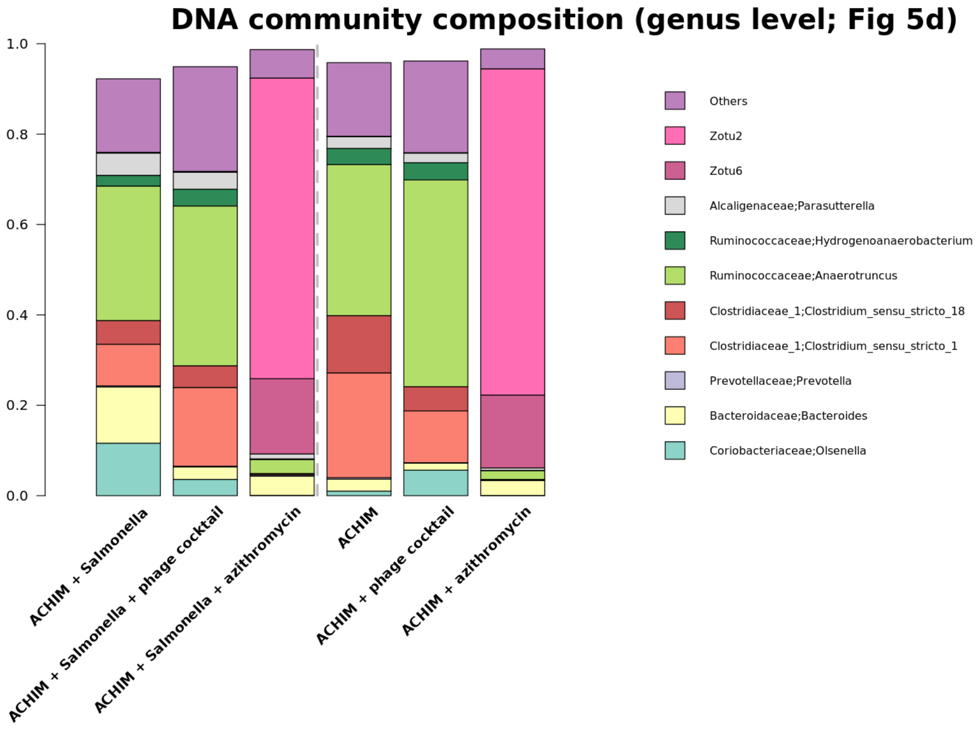
DNA community composition (genus level; Fig 5d)
1.0
0.8
0.6
0.4
0.2
0.0
ACHIM + Salmonella
ACHIM + Salmonella + phage cocktail
ACHIM + Salmonella + azithromycin
ACHIM
ACHIM + phage cocktail
ACHIM + azithromycin
Others
Zotu2
Zotu6
Alcaligenaceae;Parasutterella
Ruminococcaceae;Hydrogenoanaerobacterium
Ruminococcaceae;Anaerotruncus
Clostridiaceae_1;Clostridium_sensu_stricto_18
Clostridiaceae_1;Clostridium_sensu_stricto_1
Prevotellaceae;Prevotella
Bacteroidaceae;Bacteroides
Coriobacteriaceae;Olsenella

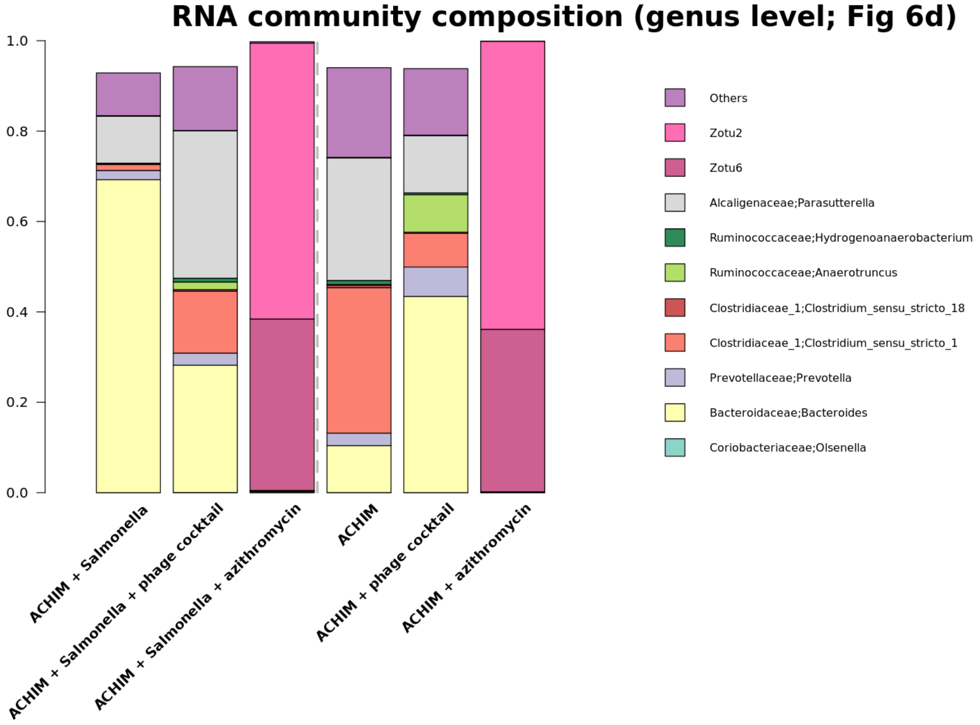
RNA community composition (genus level; Fig 6d)
1.0
0.8
0.6
0.4
0.2
0.0
ACHIM + Salmonella
ACHIM + Salmonella + phage cocktail
ACHIM + Salmonella + azithromycin
ACHIM
ACHIM + phage cocktail
ACHIM + azithromycin
Others
Zotu2
Zotu6
Alcaligenaceae;Parasutterella
Ruminococcaceae;Hydrogenoanaerobacterium
Ruminococcaceae;Anaerotruncus
Clostridiaceae_1;Clostridium_sensu_stricto_18
Clostridiaceae_1;Clostridium_sensu_stricto_1
Prevotellaceae;Prevotella
Bacteroidaceae;Bacteroides
Coriobacteriaceae;Olsenella

In [9]:

```
####################################################################################
# Calculate richness and alpha diversity (shannon indexes)

# subsampling (1000 times interation)
matr_dna_rna <- reads_16S[,c(group_dna,group_rna)]

for (i in 1:1000) {
  assign(paste("a",i,sep=""), cbind(t(rrarefy(t(matr_dna_rna), 9509))))
}

# richness calculation
OTU_freq <- list()
for (i in 1:1000){
  OTU_freq[[i]] <- as.integer(colSums(get(paste("a",i,sep="")) != 0))
}

OTU_freq_matrix <-  matrix(unlist(OTU_freq), ncol = 12, byrow = TRUE)

OTU_freq_samples <- list()
OTU_freq_mean <- c()
OTU_freq_sd <- c()
for ( i in 1:12){
  OTU_freq_samples[[i]] <- OTU_freq_matrix[,i]
  OTU_freq_mean[i] <- mean(OTU_freq_samples[[i]])
  OTU_freq_sd[i] <- sd(OTU_freq_samples[[i]])
}

# Shannon index
shannon <- list()
for (i in 1:1000){
  shannon[[i]] <- diversity(get(paste("a",i,sep="")),MARGIN=2)
}
shannon_matrix <-  matrix(unlist(shannon), ncol = 12, byrow = TRUE)

shannon_samples <- list()
shannon_mean <- c()
shannon_sd <- c()
for ( i in 1:12){
  shannon_samples[[i]] <- shannon_matrix [,i]
  shannon_mean[i] <- mean(shannon_samples[[i]])
  shannon_sd[i] <- sd(shannon_samples[[i]])
}
```

In [10]:

```
###########################################################################

# barplot for richness and shannon
greys <- c("grey0","grey60","grey50","grey80","grey40","grey55")

# DNA richness
options(repr.plot.width=14, repr.plot.height=10)
par(oma = c(2,0,0,0), mar = c(18,7,3,1))

barplot(OTU_freq_mean[group_dna],ylim = c(0,160), col = greys,border=NA, x
lab = NA , ylab = "Richness",
        xaxt="n", width = 0.8, space = 0.5, main = "DNA Richness; Fig 5a")
abline(v=3.8, col="grey", lty=2, lwd=3)
text(seq_along(sample[group_dna])*1.15, -10,
     srt = 45, adj= 1, xpd = TRUE,
     labels = label, cex=1.2, font = 2)
x = seq_along(sample[group_dna])
avg = OTU_freq_mean[group_dna]
sdev = OTU_freq_sd[group_dna] #max sdev 3.729; neglective on the plot; ski
p for ploting

# DNA shannon
barplot(shannon_mean[group_dna],ylim = c(0,4), col = greys,border=NA, xlab
 = NA , ylab = "Shannon-Wiener indexes",
        xaxt="n", width = 0.8, space = 0.5, main = "DNA Shannon-Wiener ind
exes; Fig 5b")
abline(v=3.8, col="grey", lty=2, lwd=3)
text(seq_along(sample[group_dna])*1.15, -0.1,
     srt = 45, adj= 1, xpd = TRUE,
     labels = label, cex=1.2, font = 2)
x = seq_along(sample[group_dna])
avg = shannon_mean[group_dna]
sdev = shannon_sd[group_dna] #max sdev 0.016; neglective on the plot; skip
 for ploting
```

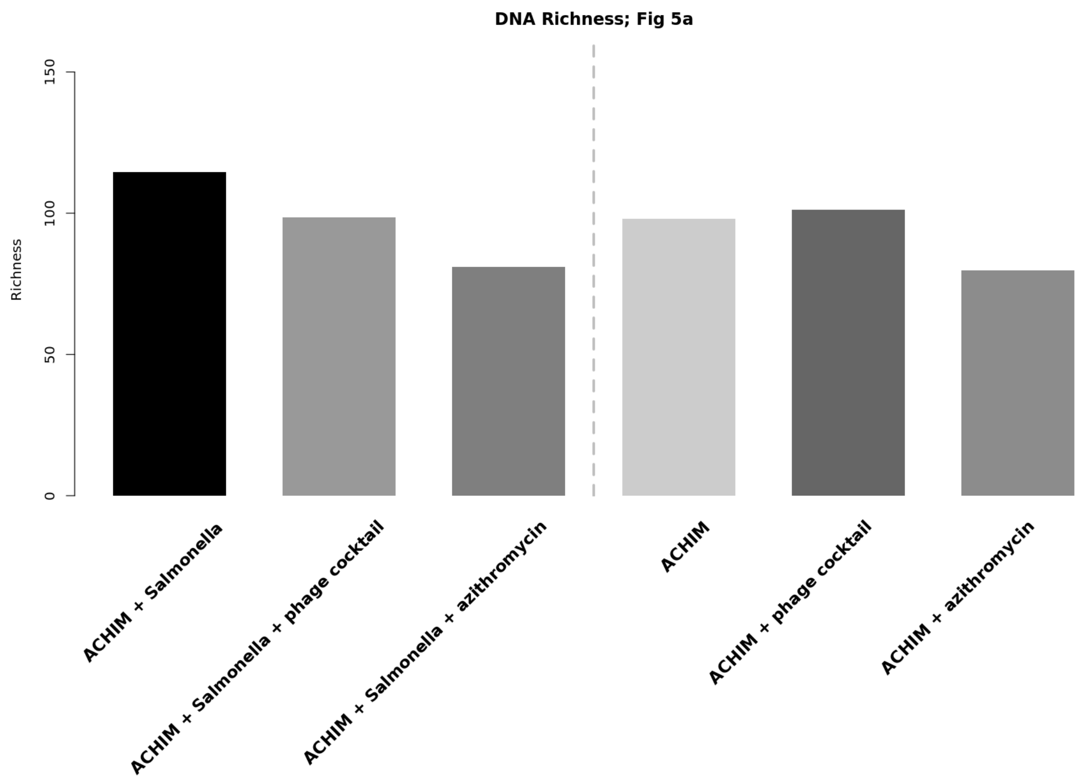
DNA Richness; Fig 5a
Richness
0
50
100
150
ACHIM + Salmonella
ACHIM + Salmonella + phage cocktail
ACHIM + Salmonella + azithromycin
ACHIM
ACHIM + phage cocktail
ACHIM + azithromycin

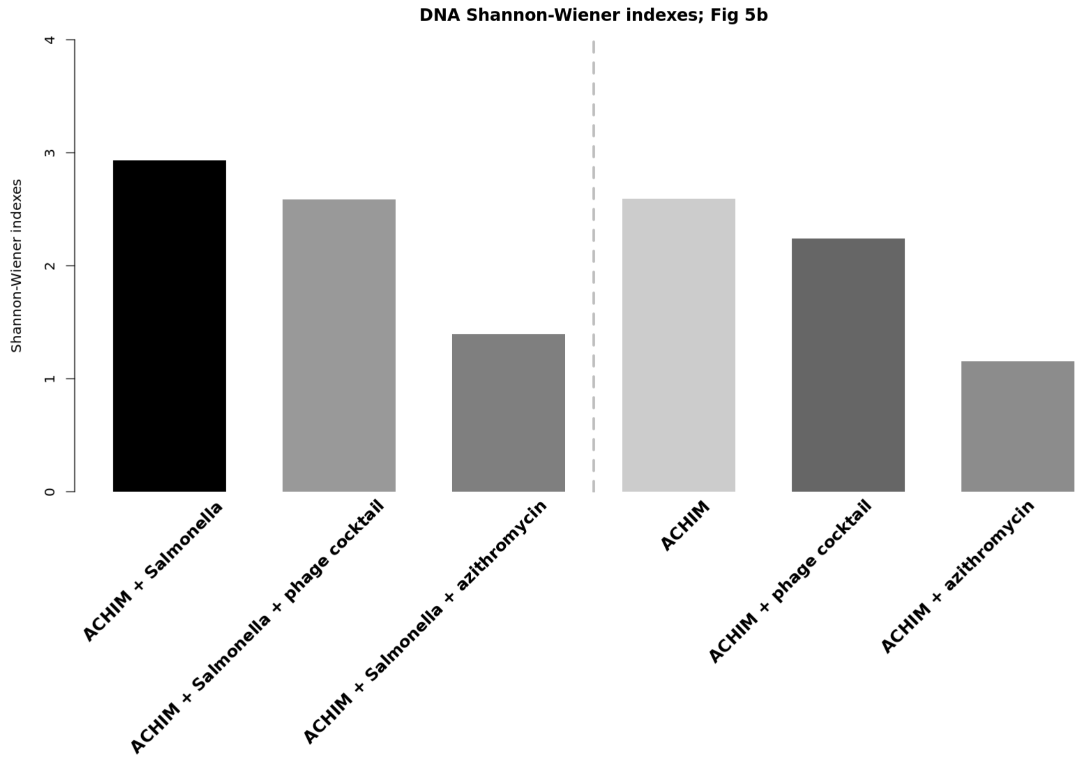
DNA Shannon-Wiener indexes; Fig 5b
Shannon-Wiener indexes
0
1
2
3
4
ACHIM + Salmonella
ACHIM + Salmonella + phage cocktail
ACHIM + Salmonella + azithromycin
ACHIM
ACHIM + phage cocktail
ACHIM + azithromycin

In [11]:

```
# RNA richness
options(repr.plot.width=14, repr.plot.height=12)
par(oma = c(2,0,0,0), mar = c(18,7,1,1))
barplot(OTU_freq_mean[group_rna],ylim = c(0,260), col = greys,border=NA, x
lab = NA , ylab = "Richness",
        xaxt="n", width = 0.8, space = 0.5, main = "RNA richness; Fig 6a")
abline(v=3.8, col="grey", lty=2, lwd=3)
text(seq_along(sample[group_rna])*1.15, -10,
     srt = 45, adj= 1, xpd = TRUE,
     labels = label, cex=1.2, font = 2)
x = seq_along(sample[group_rna])
avg = OTU_freq_mean[group_rna]
sdev = OTU_freq_sd[group_rna] #max sdev 5.01; neglective on the plot; skip
 for ploting

# RNA shannon
barplot(shannon_mean[group_rna],ylim = c(0,4), col = greys,border=NA, xlab
 = NA , ylab = "Shannon-Wiener indexes",
        xaxt="n", width = 0.8, space = 0.5, main = "RNA Shannon-Wiener ind
exes; Fig 6b")
abline(v=3.8, col="grey", lty=2, lwd=3)
text(seq_along(sample[group_rna])*1.15, -0.1,
     srt = 45, adj= 1, xpd = TRUE,
     labels = label, cex=1.2, font = 2)
x = seq_along(sample[group_rna])
avg = shannon_mean[group_rna]
sdev = shannon_sd[group_rna] #max sdev 0.017; neglective on the plot; skip
 for ploting
```

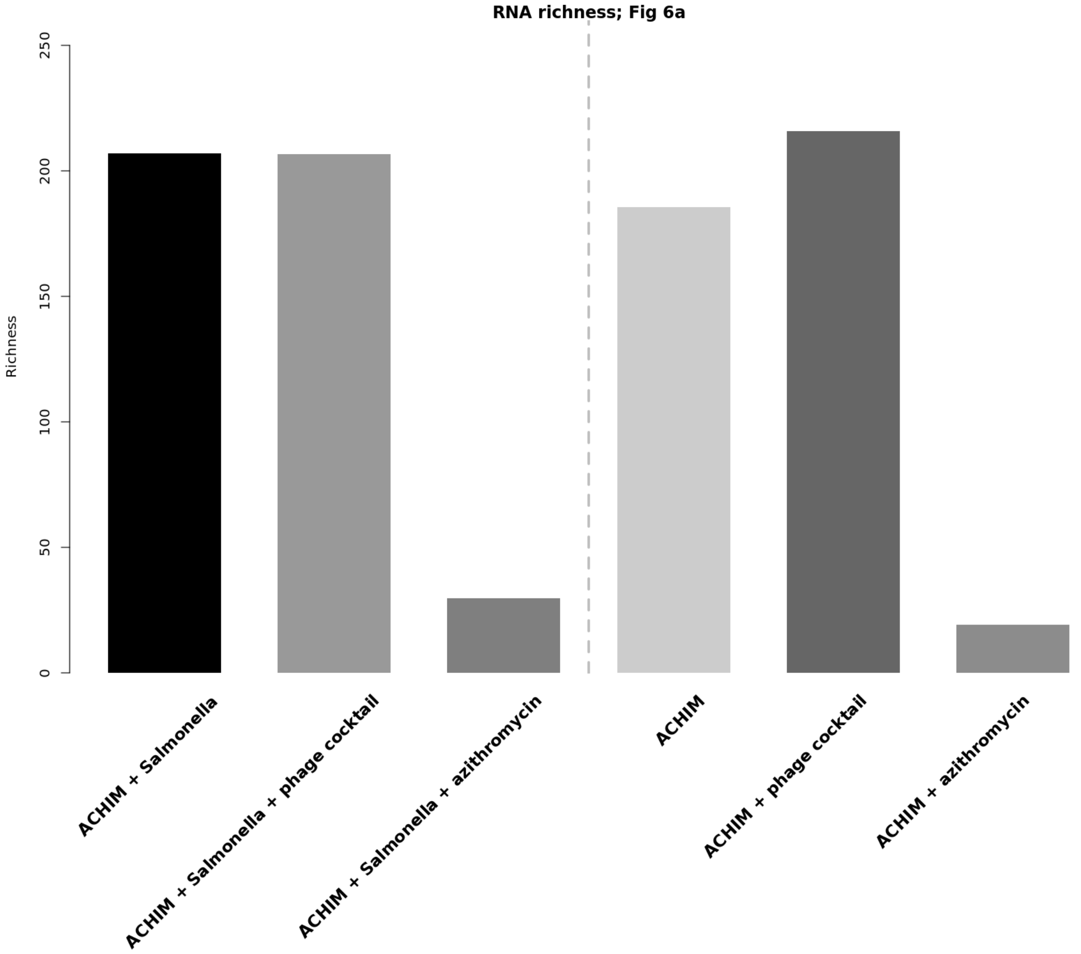
RNA richness; Fig 6a
Richness
0
50
100
150
200
250
ACHIM + Salmonella
ACHIM + Salmonella + phage cocktail
ACHIM + Salmonella + azithromycin
ACHIM
ACHIM + phage cocktail
ACHIM + azithromycin

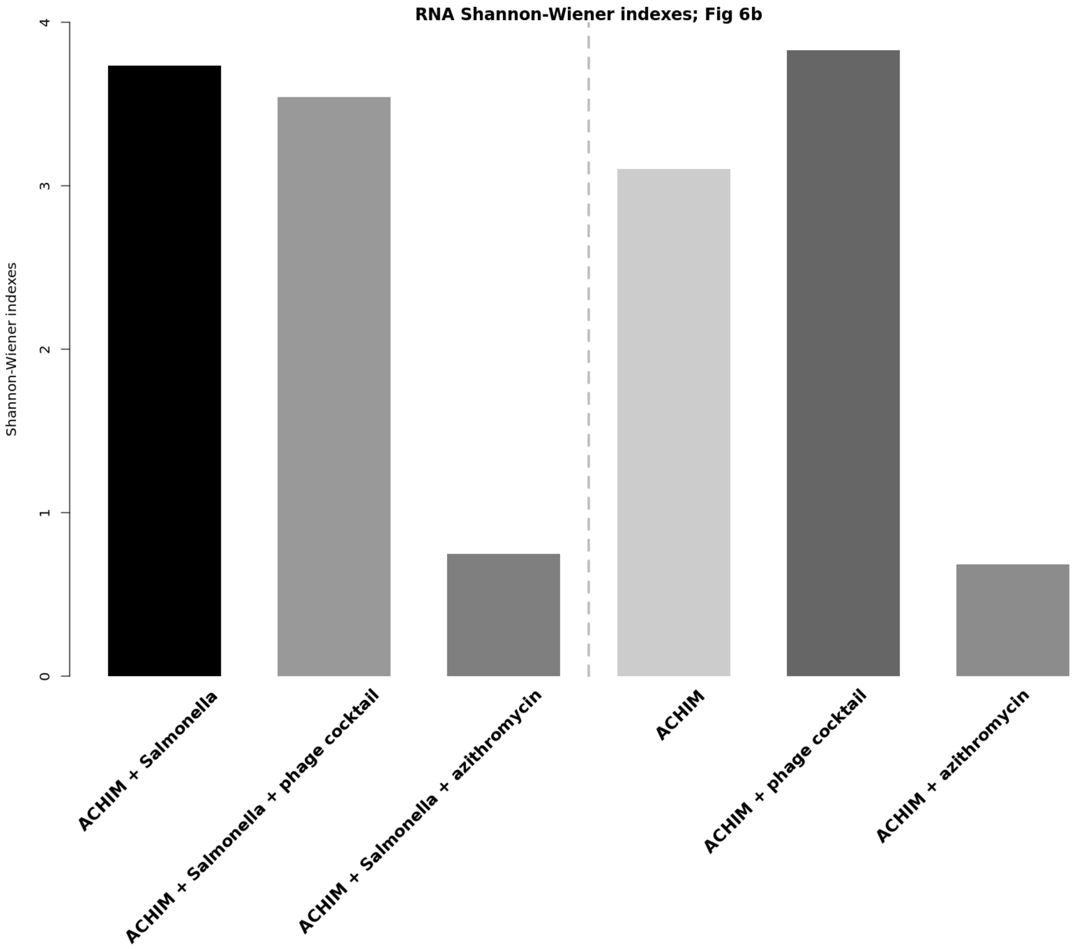
RNA Shannon-Wiener indexes; Fig 6b
Shannon-Wiener indexes
0
1
2
3
4
ACHIM + Salmonella
ACHIM + Salmonella + phage cocktail
ACHIM + Salmonella + azithromycin
ACHIM
ACHIM + phage cocktail
ACHIM + azithromycin

In [12]:
```
##########################################################
# beta diversity

# clustering
# DNA
matr <- norm_reads_16S[,group_dna]
bray_matr <- as.matrix(vegdist(t(matr), method="bray", binary=FALSE, diag=
TRUE, upper=TRUE, na.rm = FALSE))
rownames(bray_matr) <- colnames(bray_matr) <- colnames(matr)

j <- 2
distvec <- bray_matr[j:nrow(bray_matr),1]
for (i in 2:(ncol(bray_matr) - 1)) {
  j = j + 1
  distvec <- append(distvec, bray_matr[j:nrow(bray_matr),i])
}

cluster_ <- agnes(distvec, diss = TRUE, method = "complete")


options(repr.plot.width=8, repr.plot.height=8)
plot(cluster_, which.plots = 2, hang = -1, label = label ,  main = "DNA; F
ig 7b",
     axes = FALSE, xlab = "", ylab = "", sub = "")
```

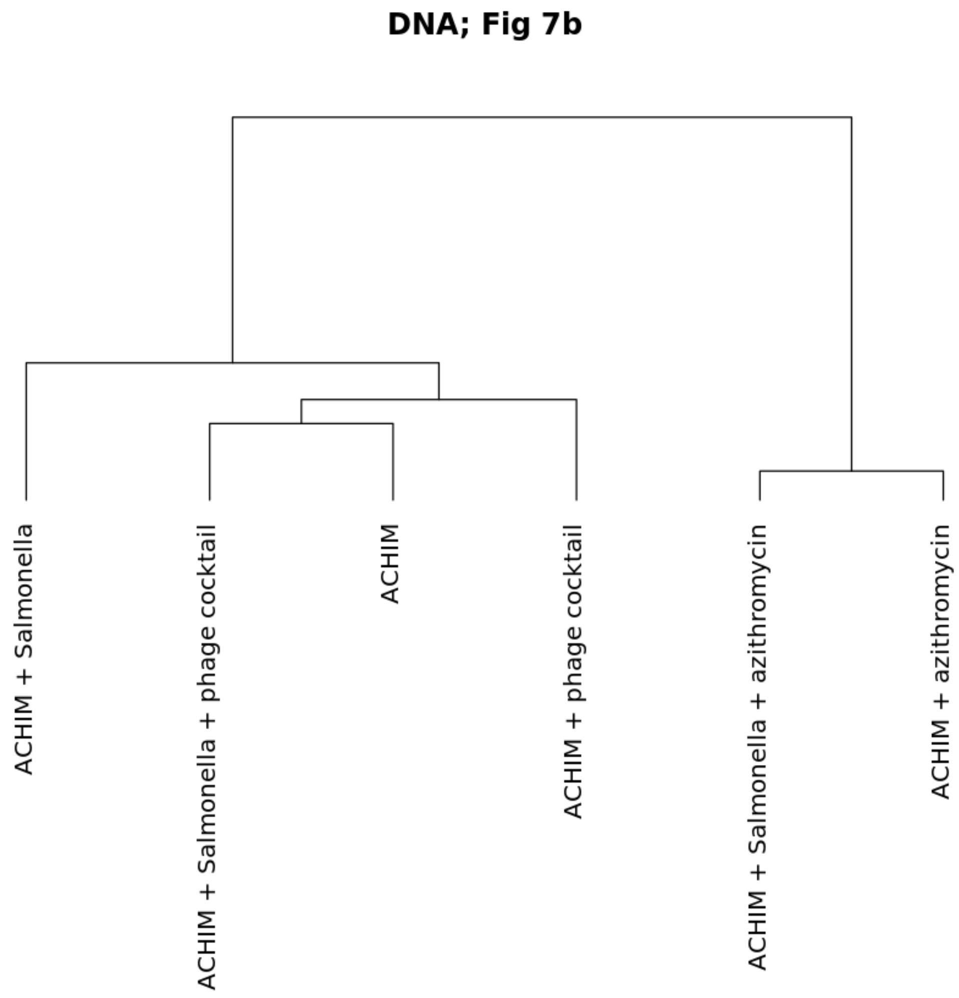

In [13]:

```
# RNA
matr <- norm_reads_16S[,group_rna]
bray_matr <- as.matrix(vegdist(t(matr), method="bray", binary=FALSE, diag=
TRUE, upper=TRUE, na.rm = FALSE))
rownames(bray_matr) <- colnames(bray_matr) <- colnames(matr)

j <- 2
distvec <- bray_matr[j:nrow(bray_matr),1]
for (i in 2:(ncol(bray_matr) - 1)) {
  j = j + 1
  distvec <- append(distvec, bray_matr[j:nrow(bray_matr),i])
}


cluster_ <- agnes(distvec, diss = TRUE, method = "complete")

options(repr.plot.width=8, repr.plot.height=8)
plot(cluster_, which.plots = 2, hang = -1, label = label ,  main = "RNA; F
ig 7c",
     axes = FALSE, xlab = "", ylab = "", sub = "")
```

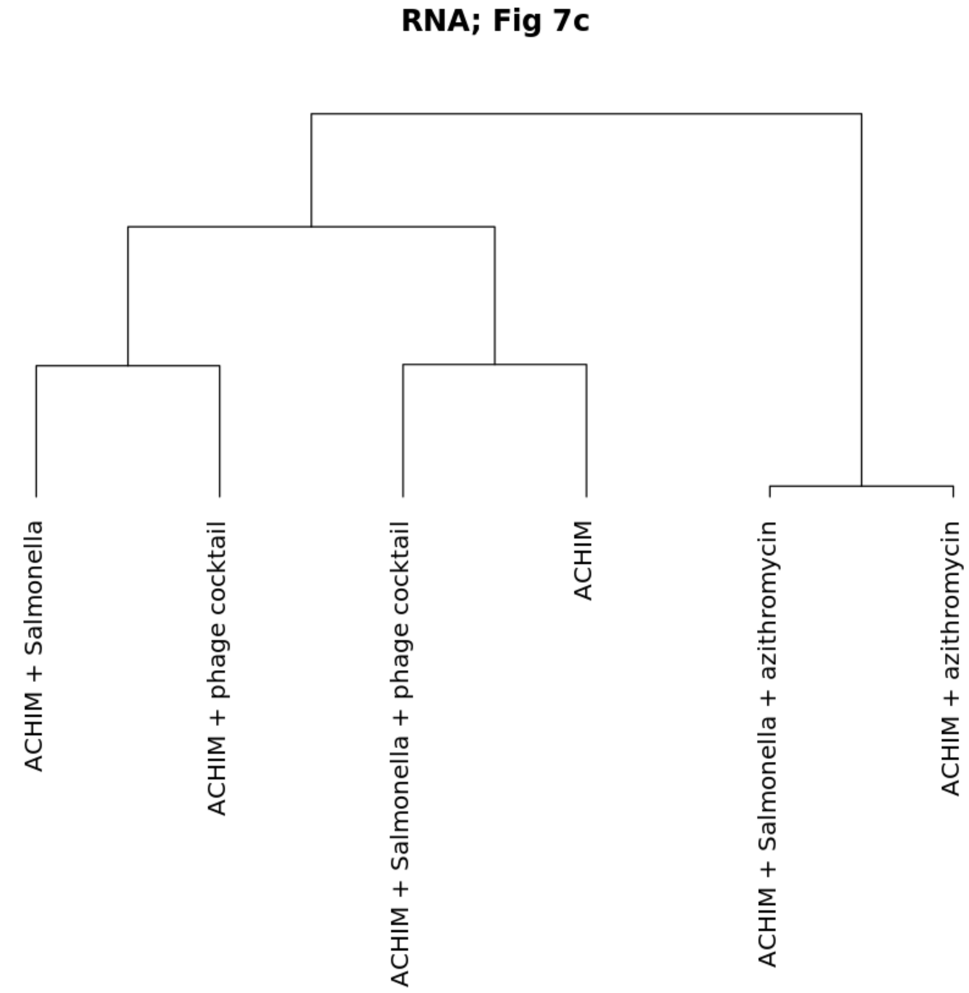

In [14]:

```
# PCoA
matr <- norm_reads_16S[,group]
bray_matr <- as.matrix(vegdist(t(matr), method="bray", binary=FALSE, diag=
TRUE, upper=TRUE, na.rm = FALSE))
colnames(bray_matr) <- rownames(bray_matr) <- sample[group]


pcoa <- pcoa(bray_matr,correction = "cailliez")
pcoa$values[c(1,2),3]
xlab = paste("PC1 (", 100*round(pcoa$values[1,3], 2), "%)", sep = "")
ylab = paste("PC2 (", 100*round(pcoa$values[2,3], 2), "%)", sep = "")

color <- c()
Sal <- grep("Salmonella",sample[group])
nonSal <- setdiff(group,Sal)
color[Sal] <-  c(brewer.pal(3,"Reds"),brewer.pal(3,"Reds"))
color[nonSal] <-  c(c("grey80","grey50","black"),c("grey80","grey50","blac
k"))

pch <- c()
pch[group_dna] <- 17
pch[group_rna] <- 25

options(repr.plot.width=8, repr.plot.height=8)
plot(pcoa$vectors[,1], pcoa$vectors[,2], cex = 2.5, xlab = xlab, ylab = yl
ab,
     xlim = c(-0.6,0.7), ylim = c(-0.6,0.7), pch = pch, col = color, main
= "PCoA; Fig 7a")
text(0.18, rev(seq(0.3,0.7,0.07)), labels = label, cex = 0.5, pos = 4, adj
 = 1)
text(c(0.03,0.1), 0.72, labels = c("DNA","RNA"), cex = 0.5, pos = 4, adj =
 1)

points(replicate(6,0.1), rev(seq(0.3,0.7,0.07)), pch = 17, col= color[1:6
])
points(replicate(6,0.15), rev(seq(0.31,0.71,0.07)), pch = 25, col= color[1
:6])
```

0.567448299243685   0.267777834306916

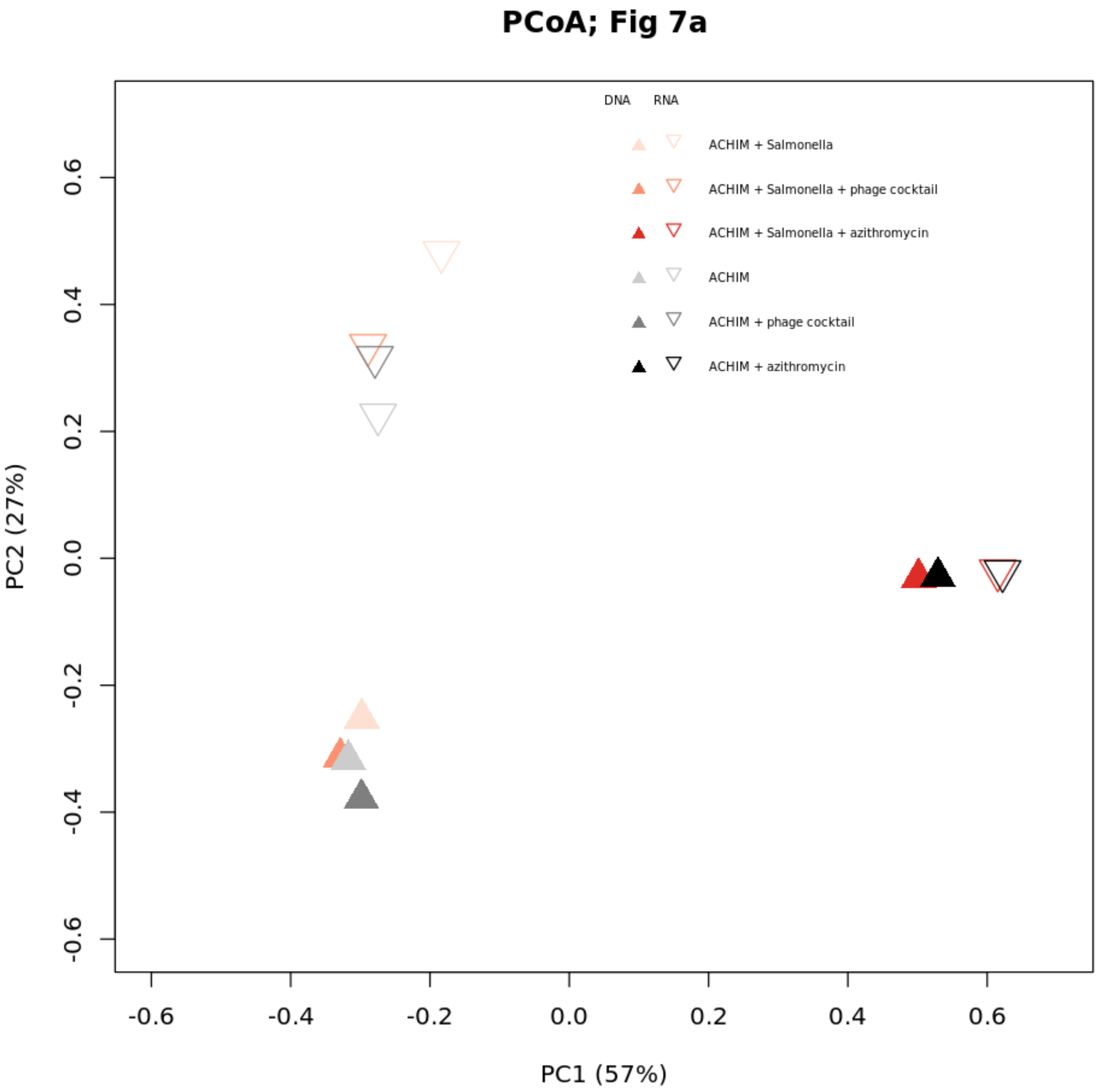

In [15]:
```
# heatmap at OTU level
# make Fig S3

# sort the sequence table (Supplementary_Table_2), sort the rows by taxa,
 and add R color keys for phylums
sOTU_matr<- read.delim(paste0(path,"Supplementary_table_3.tsv"), sep =
"\t", header = T)

sOTU_id <- as.matrix(sOTU_matr$sub_seq_id)
sOTU_taxa <- as.matrix(sOTU_matr$subtaxa)
sOTU_phyta <- as.matrix(sOTU_matr$phyla)
sOTU_reads <- as.matrix(sOTU_matr[2:(ncol(sOTU_matr)-3)])
color <- as.matrix(sOTU_matr$colors)

unique(sOTU_matr$phyla)
rownames(as.matrix(table(sOTU_phyta)))
a <- as.matrix(table(sOTU_phyta)[match(unique(sOTU_matr$phyla),rownames(a
s.matrix(table(sOTU_phyta))))])
b <- color[match(unique(sOTU_matr$phyla),sOTU_matr$phyla)]

# the color key bar for families
options(repr.plot.width=8, repr.plot.height=8)
barplot(cbind(a,0), col = b,legend=T, las =2, cex.names = 0.7,ylim = c(0,2
29),xlim = c(0,50),border=NA)

# pheatmap
options(repr.plot.width=6, repr.plot.height=18)
submatr_ <- sOTU_reads[nrow(sOTU_reads):1,]
rownames(submatr_) <- gsub("_", "",sOTU_id[nrow(sOTU_reads):1])
pheatmap(submatr_, cluster_cols = F, cluster_rows = F, border_color = "whi
te", color = c("white",colorRampPalette(brewer.pal(n = 9, name = "Reds"))(
6894)), cex =0.7 )

## integrate the color key bar and the heatmap by using Adobe Illustrator
```

Coriobacteriaceae Other_bacteroidetes Bacteroidaceae Prevotellaceae Other_firmicutes Clostridiaceae_1 Lachnospiraceae Peptostreptococcaceae Ruminococcaceae Veillonellaceae Other_proteobacteria Alcaligenaceae Other_bacteria

'Alcaligenaceae' 'Bacteroidaceae' 'Clostridiaceae_1' 'Coriobacteriaceae' 'Lachnospiraceae' 'Other_bacteria' 'Other_bacteroidetes' 'Other_firmicutes' 'Other_proteobacteria' 'Peptostreptococcaceae' 'Prevotellaceae' 'Ruminococcaceae' 'Veillonellaceae'

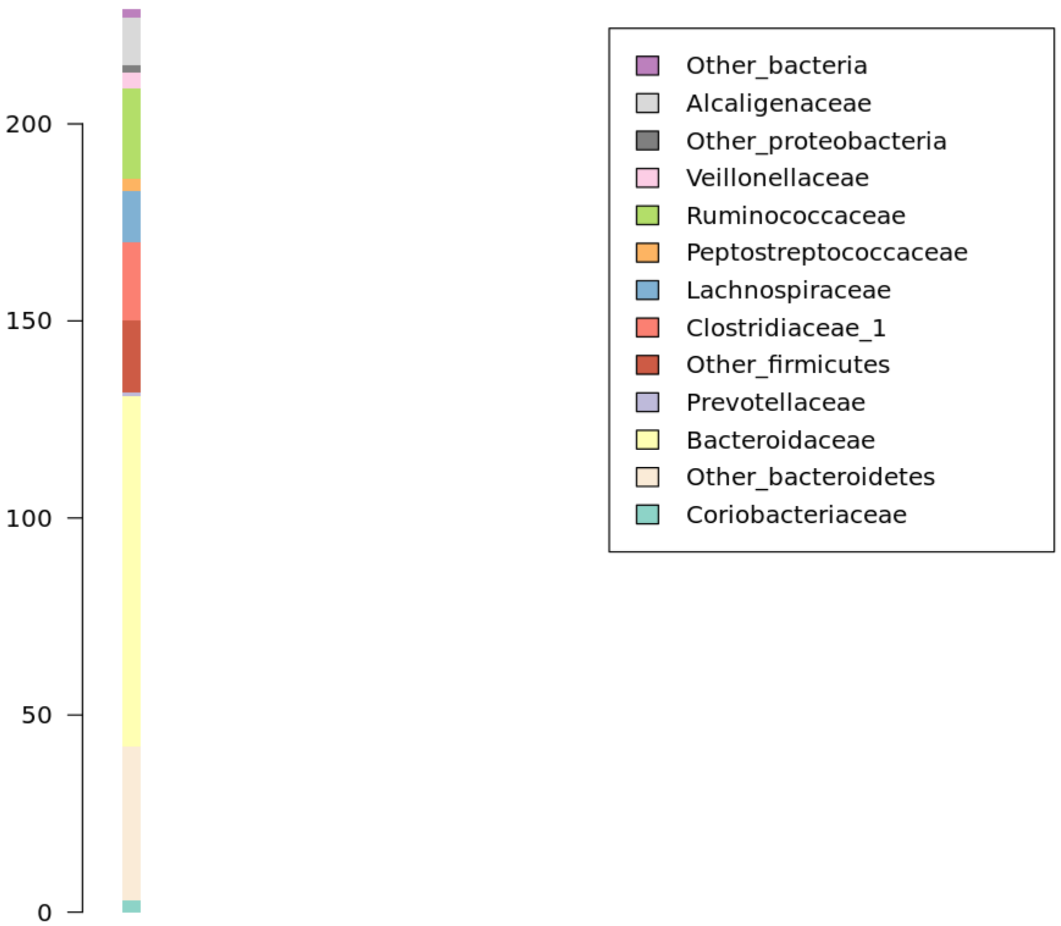

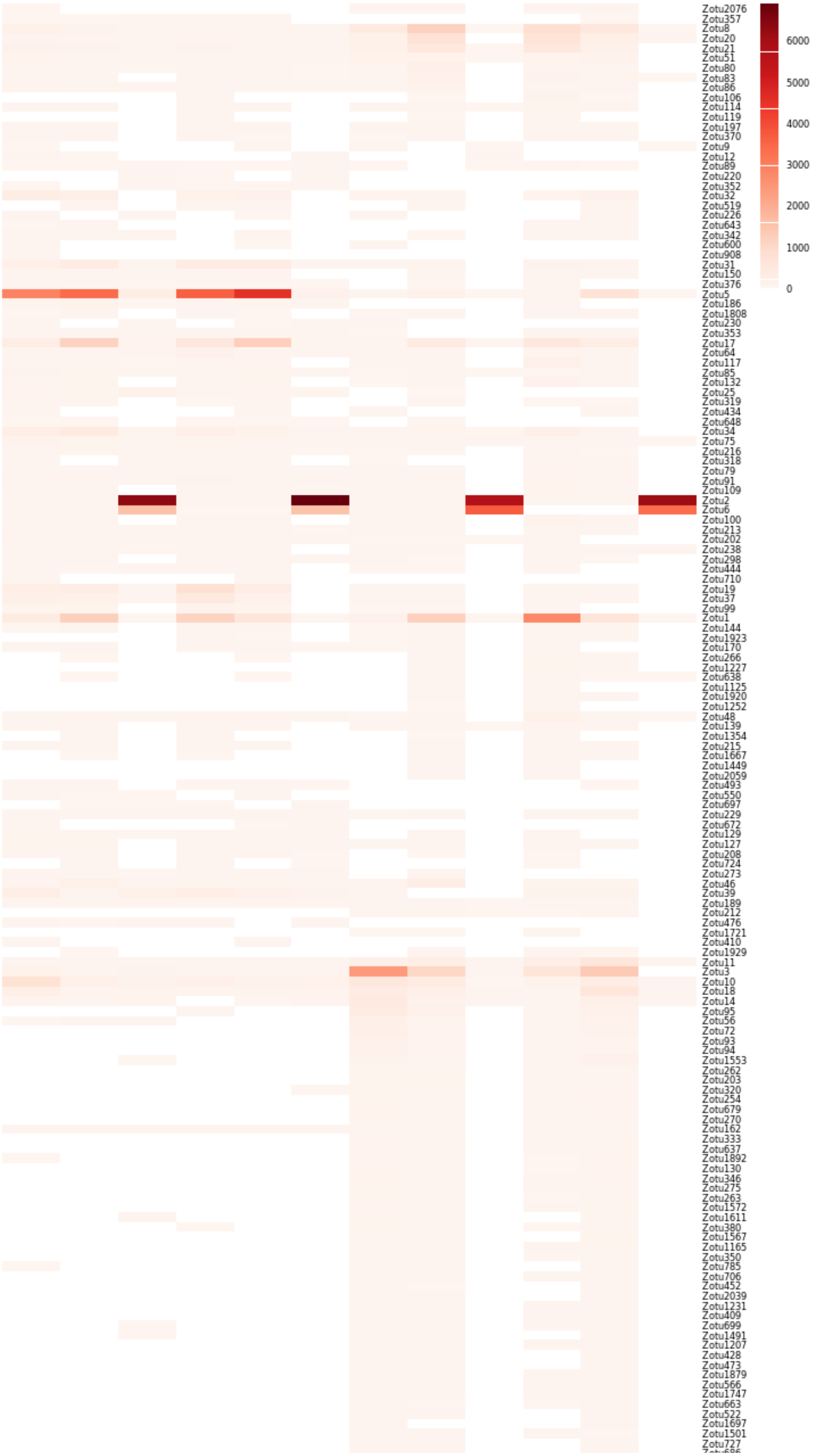
Zotu2076
Zotu357
Zotu8
Zotu20
Zotu21
Zotu51
Zotu80
Zotu83
Zotu86
Zotu106
Zotu114
Zotu119
Zotu197
Zotu370
Zotu9
Zotu12
Zotu89
Zotu220
Zotu352
Zotu32
Zotu519
Zotu226
Zotu643
Zotu342
Zotu600
Zotu908
Zotu31
Zotu150
Zotu376
Zotu5
Zotu186
Zotu1808
Zotu230
Zotu353
Zotu17
Zotu64
Zotu117
Zotu85
Zotu132
Zotu25
Zotu319
Zotu434
Zotu648
Zotu34
Zotu75
Zotu216
Zotu318
Zotu79
Zotu91
Zotu109
Zotu2
Zotu6
Zotu100
Zotu213
Zotu202
Zotu238
Zotu298
Zotu444
Zotu710
Zotu19
Zotu37
Zotu99
Zotu1
Zotu144
Zotu1923
Zotu170
Zotu266
Zotu1227
Zotu638
Zotu1125
Zotu1920
Zotu1252
Zotu48
Zotu139
Zotu1354
Zotu215
Zotu1667
Zotu1449
Zotu2059
Zotu493
Zotu550
Zotu697
Zotu229
Zotu672
Zotu129
Zotu127
Zotu208
Zotu724
Zotu273
Zotu46
Zotu39
Zotu189
Zotu212
Zotu476
Zotu1721
Zotu410
Zotu1929
Zotu11
Zotu3
Zotu10
Zotu18
Zotu14
Zotu95
Zotu56
Zotu72
Zotu93
Zotu94
Zotu1553
Zotu262
Zotu203
Zotu320
Zotu254
Zotu679
Zotu270
Zotu162
Zotu333
Zotu637
Zotu1892
Zotu130
Zotu346
Zotu275
Zotu263
Zotu1572
Zotu1611
Zotu380
Zotu1567
Zotu1165
Zotu350
Zotu785
Zotu706
Zotu452
Zotu2039
Zotu1231
Zotu409
Zotu699
Zotu1491
Zotu1207
Zotu428
Zotu473
Zotu1879
Zotu566
Zotu1747
Zotu663
Zotu522
Zotu1697
Zotu1501
Zotu727
6000
5000
4000
3000
2000
1000
0

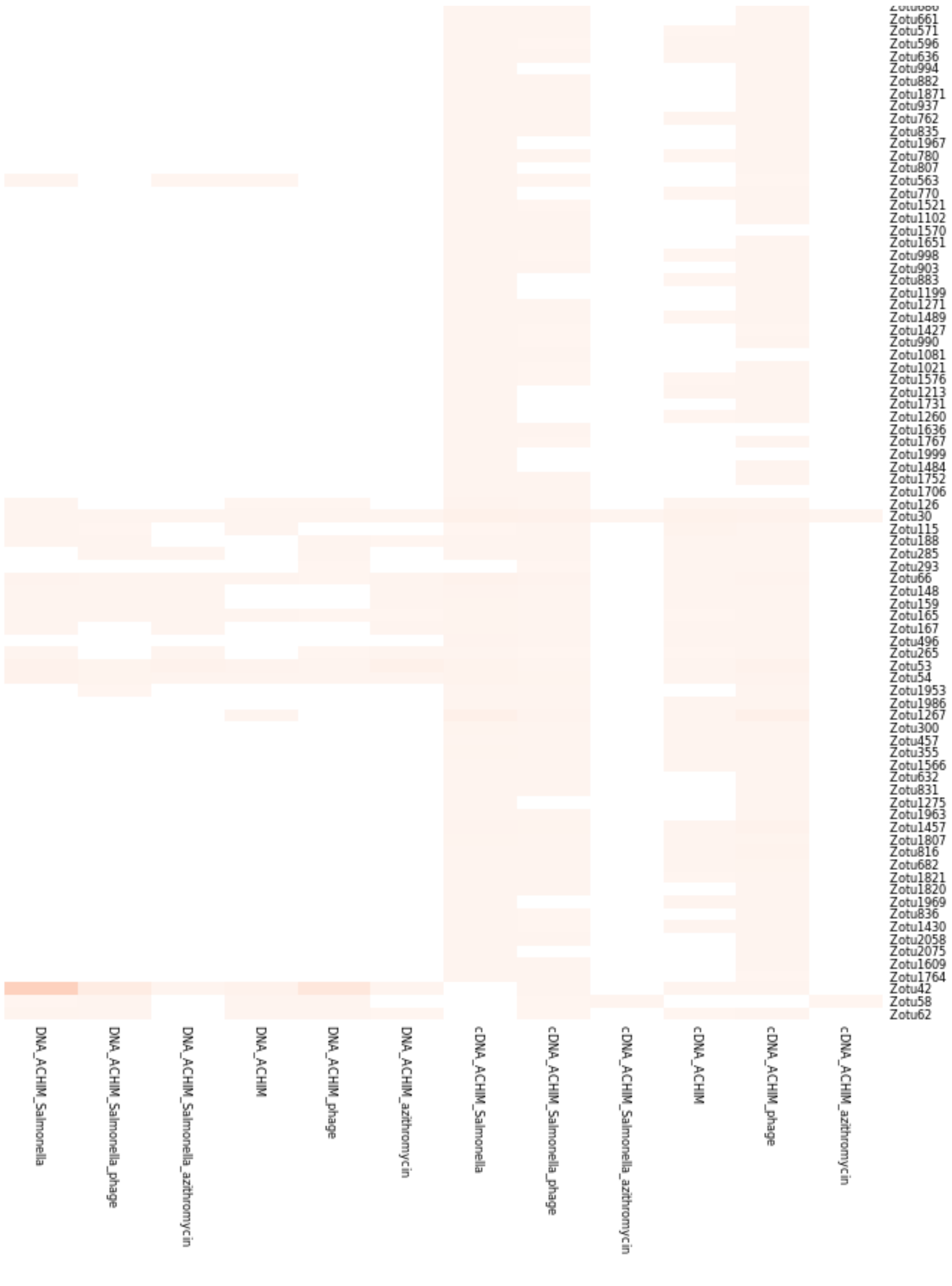
Zotu661
Zotu571
Zotu596
Zotu636
Zotu994
Zotu882
Zotu1871
Zotu937
Zotu762
Zotu835
Zotu1967
Zotu780
Zotu807
Zotu563
Zotu770
Zotu1521
Zotu1102
Zotu1570
Zotu1651
Zotu998
Zotu903
Zotu883
Zotu1199
Zotu1271
Zotu1489
Zotu1427
Zotu990
Zotu1081
Zotu1021
Zotu1576
Zotu1213
Zotu1731
Zotu1260
Zotu1636
Zotu1767
Zotu1999
Zotu1484
Zotu1752
Zotu1706
Zotu126
Zotu30
Zotu115
Zotu188
Zotu285
Zotu293
Zotu66
Zotu148
Zotu159
Zotu165
Zotu167
Zotu496
Zotu265
Zotu53
Zotu54
Zotu1953
Zotu1986
Zotu1267
Zotu300
Zotu457
Zotu355
Zotu1566
Zotu632
Zotu831
Zotu1275
Zotu1963
Zotu1457
Zotu1807
Zotu816
Zotu682
Zotu1821
Zotu1820
Zotu1969
Zotu836
Zotu1430
Zotu2058
Zotu2075
Zotu1609
Zotu1764
Zotu42
Zotu58
Zotu62
DNA_ACHIM_Salmonella
DNA_ACHIM_Salmonella_phage
DNA_ACHIM_Salmonella_azithromycin
DNA_ACHIM
DNA_ACHIM_phage
DNA_ACHIM_azithromycin
cDNA_ACHIM_Salmonella
cDNA_ACHIM_Salmonella_phage
cDNA_ACHIM_Salmonella_azithromycin
cDNA_ACHIM
cDNA_ACHIM_phage
cDNA_ACHIM_azithromycin
